# Risk of COVID-19 hospitalizations among school-aged children in Scotland: A national incident cohort study

**DOI:** 10.7189/jogh.12.05044

**Published:** 2022-09-23

**Authors:** Ting Shi, Jiafeng Pan, Emily Moore, Srinivasa Vittal Katikireddi, Annemarie B Docherty, Lynda Fenton, Colin McCowan, Utkarsh Agrawal, Steven Kerr, Syed Ahmar Shah, Sarah J Stock, Colin R Simpson, Chris Robertson, Aziz Sheikh, Public Health Scotland and the EAVE II Collaborators

**Affiliations:** 1 Usher Institute, Edinburgh Medical School, University of Edinburgh, Edinburgh, Scotland, UK.; 2 Department of Mathematics and Statistics, University of Strathclyde, Glasgow, Scotland, UK.; 3 Public Health Scotland, Glasgow, Scotland, UK.; 4 MRC/CSO Social & Public Health Sciences Unit, University of Glasgow, Glasgow, Scotland, UK.; 5 School of Medicine, University of St Andrews, St Andrews, Scotland, UK.; 6 School of Health, Wellington Faculty of Health, Victoria University of Wellington, Wellington, New Zealand

## Abstract

**Background:**

There is considerable policy, clinical and public interest about whether children should be vaccinated against SARS-CoV-2 and, if so, which children should be prioritised (particularly if vaccine resources are limited). To inform such deliberations, we sought to identify children and young people at highest risk of hospitalization from COVID-19.

**Methods:**

We used the Early Pandemic Evaluation and Enhanced Surveillance of COVID-19 (EAVE II) platform to undertake a national incident cohort analysis to investigate the risk of hospitalization among 5-17 years old living in Scotland in risk groups defined by the living risk prediction algorithm (QCOVID). A Cox proportional hazard model was used to derive hazard ratios (HR) and 95% confidence intervals (CIs) for the association between risk groups and COVID-19 hospital admission. Adjustments were made for age, sex, socioeconomic status, co-morbidity, and prior hospitalization.

**Results:**

Between March 1, 2020 and November 22, 2021, there were 146 183 (19.4% of all 752 867 children in Scotland) polymerase chain reaction (PCR) confirmed SARS-CoV-2 infections among 5-17 years old. Of those with confirmed infection, 973 (0.7%) were admitted to hospital with COVID-19. The rate of COVID-19 hospitalization was higher in those within each QCOVID risk group compared to those without the condition. Similar results were found in age stratified analyses (5-11 and 12-17 years old). Risk groups associated with an increased risk of COVID-19 hospital admission, included (adjusted HR, 95% CIs): sickle cell disease 14.35 (8.48-24.28), chronic kidney disease 11.34 (4.61-27.87), blood cancer 6.32 (3.24-12.35), rare pulmonary diseases 5.04 (2.58-9.86), type 2 diabetes 3.04 (1.34-6.92), epilepsy 2.54 (1.69-3.81), type 1 diabetes 2.48 (1.47-4.16), Down syndrome 2.45 (0.96-6.25), cerebral palsy 2.37 (1.26-4.47), severe mental illness 1.43 (0.63-3.24), fracture 1.41 (1.02-1.95), congenital heart disease 1.35 (0.82-2.23), asthma 1.28 (1.06-1.55), and learning disability (excluding Down syndrome) 1.08 (0.82-1.42), when compared to those without these conditions. Although our Cox models were adjusted for a number of potential confounders, residual confounding remains a possibility.

**Conclusions:**

In this national study, we observed an increased risk of COVID-19 hospital admissions among school-aged children with specific underlying long-term health conditions compared with children without these conditions.

There is considerable policy, clinical and public interest about whether children should be vaccinated against SARS-CoV-2 and, if so, whether some children should be prioritised for vaccination. Meanwhile, there is considerable uncertainty about the benefits and risks of COVID-19 vaccines in children, as well as concerns about limited vaccine supplies in many parts of the world.

A number of countries are now administering COVID-19 vaccines to children and young people. In the United States (US), the Food and Drug Administration (FDA) authorised Pfizer-BioNTech COVID-19 vaccine for use in adolescents aged 16-17 years old on December 11, 2020, then extended the roll-out of vaccines to 12-15 years old on May 10, 2021 [1], and now everyone aged 5 and older is recommended to get a COVID-19 vaccine [2]. The European Medicines Agency (EMA) announced on July 23, 2021 that the Moderna vaccine can be used in children aged 12-17 years [3]. Thus far, 20 European countries are either currently vaccinating or planning to roll out their vaccination programmes to children aged 12 and over, alongside Canada, China, Israel, Japan, Philippines, Singapore, the United Arab Emirates (UAE) and the US.

In contrast, the United Kingdom’s (UK) Joint Committee on Vaccination and Immunisation (JCVI) has not recommended the blanket vaccination of all children and young people. Rather, at the time of this analysis, the JCVI’s policy was to recommend COVID-19 vaccination to all children aged 16-17 years and to offer vaccines to 12-15 years old with underlying conditions that put them at increased risk of serious COVID-10 outcomes [4,5]. This was then over-ruled following a request by the Secretary of State for Health and Social Care who asked the UK’s Chief Medical Officers to consider wider societal implications of vaccination, which led to a decision to extend the offer of vaccinations to all children aged 12-15 years [6]. The existing evidence base on which children are at highest risk of COVID-19 hospitalizations is however limited and difficult to interpret as there have been very few population-based analyses [7-9].

Up to December 5, 2021, the percentage of population aged 5-17 years old vaccinated with first dose, second dose and booster or third dose was 34.1%, 6.9% and 0.4%, respectively. In order to inform these key national deliberations on vaccine prioritization, we sought to identify school-aged children with certain conditions that put them at increased risk of COVID-19 hospitalization and deaths compared with children without the same conditions.

## METHODS

We have published a general protocol for Early Pandemic Evaluation and Enhanced Surveillance of COVID-19 (EAVE II) and cohort profile [10,11]. We did not however have a specific analysis plan for this pediatric work as this was an urgently requested analysis by the JCVI. Thus, we described the analysis plan in detail as below.

### Study design

EAVE II is a Scotland-wide COVID-19 surveillance platform that has been used to track and forecast the epidemiology of COVID-19, inform risk stratification assessment in adults, and investigate vaccine effectiveness and safety [10-13]. The EAVE II platform comprises of national health care data sets on 5.4 million people ( ~ 99% of the Scottish population) deterministically linked through the Community Health Index (CHI) number, which is a unique identifier used in all health care contacts across NHS Scotland.

We used the EAVE II platform to describe the demographic profile of school-aged children with SARS-CoV-2 infections and COVID-19 hospital admissions. We also undertook a national incident cohort analysis to investigate risks of hospitalization in pre-defined risk groups. The cohort was set up on March 1, 2020. This analysis was based upon all 752 867 children and young people in the EAVE II linked data set aged 5-17 years on March 1, 2020.

### Data sources

The national data sets linked using CHI number were the Electronic Communication of Surveillance in Scotland (national database for all virology testing), primary care (demographics and clinical history), the Scottish Morbidity Record (which records hospitalizations), National Records of Scotland (which records mortality data), and Prescribing Information System (for prescription data). A data linkage diagram is available in Figure S1 in the **Online Supplementary Document**.

### Outcomes

Building on methods that have previously been described in detail [13,14]. we defined individuals who tested positive with real-time reverse transcription-polymerase chain reaction (RT-PCR) as having SARS-CoV-2 infections. We defined a COVID-19 hospital admission as being hospitalised within 28 days following a positive RT-PCR test for SARS-CoV-2, including those who tested positive while hospitalised, or those who were hospitalised with an admission diagnosis of COVID-19 (Table S1 in the **Online Supplementary Document**). We also report results using restrictions to this definition for more specificity – admission within 14 days and to only consider admissions where COVID-19 was listed as an admission diagnosis. COVID-19 related deaths were all-cause deaths occurring within 28 days after a positive test for SARS-CoV-2 that were registered with National Records Scotland and included death certification, or deaths with COVID-19 on the death certificate as the cause of death.

### Risk groups

The risk groups were measured on March 1, 2020 and defined by the living risk prediction algorithm (QCOVID), which consists of 30 clinical characteristics identified from primary care records that are known to be associated with increased risk of serious COVID-19 outcomes in adults (Box S1 in the **Online Supplementary Document**) [15]. QCOVID was based on GP Read codes with derived variables created for broader diagnostic coding conditions [16]. We excluded risk groups that were either not relevant to the pediatric population (ie, care home/homeless, chronic obstructive pulmonary disease, coronary heart disease, dementia, Parkinson disease) or had substantial missing data (body mass index (BMI), ethnicity; percentage of missing data available in Table S2 in the **Online Supplementary Document**). We only included the pre-defined risk group if there were at least five COVID-19 hospitalizations within a risk group during the study period. This resulted in a total of 13 risk groups being included and analyzed, namely: asthma, blood cancer, cerebral palsy, chronic kidney disease, congenital heart disease, type 1 diabetes, type 2 diabetes, epilepsy, learning disability, fracture, rare pulmonary diseases, severe mental illness, and sickle cell disease (Table S2 in the **Online Supplementary Document**).

### Statistical analysis

Our analysis covered the period from March 1, 2020 to November 22, 2021. All individuals were followed from March 1, 2020 until the date of COVID-19 related hospitalization, date of death or censor date (November 22, 2021) whichever came first.

A Cox proportional hazard model was used to derive hazard ratios (HRs) and 95% confidence intervals (CIs) for the association between each risk group and COVID-19 hospital admissions. This model, with calendar time as the timescale, eliminates the need to model the underlying temporal trends, which are estimated as the baseline hazard. The Cox model adjusted for the risk factors that were likely to be associated with COVID-19 hospitalizations, namely a spline of age, sex, socioeconomic status, risk groups of interest, and number of prior all-cause hospitalization in the past two years from March 1, 2020. Socioeconomic status was determined on the basis of the Scottish Index of Multiple Deprivation (SIMD). The SIMD classification is based on deprivation quintiles: quintile 1 refers to the most deprived and quintile 5 refers to the most affluent. SIMD was assigned according to residential postcode. Prior hospitalization adjustment was used as a marker of severity and/or health care seeking behavior. Similarly, the Cox proportional hazard models were fitted to estimate the association between each risk group and the outcomes of being tested and having a positive test for SARS-CoV-2 infection separately. All analyses were stratified by age group (5-11 years vs 12-17 years). At the request of peer-reviewers, we have also investigated if the magnitude of the differences in risk of COVID-19 hospitalization comparing those with the conditions of interest and those without varied across the period of the pandemic with different variants (Wild type: March 1, 2020 to January 4, 2021; Alpha variant dominant: January 5, 2021 to May 16, 2021; and Delta variant dominant: May 17, 2021 to the end of study period) by fitting the Cox model in each period, respectively.

Both the Cox proportional hazards model and estimation of cumulative incidence (Figure S2 in the **Online Supplementary Document**) used sampling weights, which were used to correct for the size of the registered general practice population being greater than the population in Scotland (some due to individuals who had recently moved). These weights were derived by matching the age and sex numbers in the general practice data to the Scottish population data. This adjustment ensured that the denominators in the tables matched the Scottish population.

Analyses were carried out in R (version 3.6.1).

### Use of reporting guideline

We followed the STrengthening the Reporting of OBservational studies in Epidemiology (STROBE) checklist [17] to guide transparent reporting of this cohort study (Table S3 in the **Online Supplementary Document**).

## RESULTS

There were in total 752 867 children aged 5-17 years old included in this analysis. 117 487 had at least one risk group of interest and 10 907 had more than one risk group. The median follow-up was 20.7 (range 0-20.7) months for COVID-19 hospitalization. Among them, there were 146 183 (19.4% of all children) confirmed SARS-CoV-2 infections, of whom 973 (0.7%) were admitted to hospitals for COVID-19 (of whom, 333 tested positive on the day of admission or after admission to hospital, Table S9 in the **Online Supplementary Document**). COVID-19 was listed as a reason for admission in 452/973 children, including 269 in whom it was the main reason for admission. The following analyses were based on 973 children. The median length of stay for the COVID-19 admissions was 1 day with an interquartile range (IQR) of (0,2) days. However, the number of COVID-19 related deaths was too small (<5) to enable us carry out any analysis, so we did not include them in this study. The baseline characteristics for each risk group of interest are available in Table S4 in the **Online Supplementary Document**. The most common risk conditions were asthma, learning disability (excluding Downs syndrome) and fracture. A flowchart of study population is available in Figure S3 in the **Online Supplementary Document**.

The rate of being tested and COVID-19 hospitalization was consistently higher within each risk group compared to those without the condition (**Table 1**). Similar results were found in the age-stratified analysis (5-11 years vs 12-17 years) or when age group was included as a continuous variable (Figure S4 in the **Online Supplementary Document**). The proportion of hospital admissions among those who tested positive was also consistently higher within each risk group of interest (**Table 1**). However, the difference in the rate of testing positive with SARS-CoV-2 varied in different directions among school-aged children with or without the risk group (**Table 1**).

**Table 1:** Number and rate (per 100 000 persons) of being tested, testing positive and COVID-19 hospitalization respectively in each risk group of interest among children and young people aged 5 to 17 years old

Risk group	Overall number	Number of being tested; n (rate per 100 000)	Number of testing positive with SARS-CoV-2; n (rate per 100 000)	Number of hospitalization with COVID-19 ; n (rate per 100 000)	Percentage of hospitalization among testing positive (%)
Asthma – No	689 404	420 478 (60991.5)	133 199 (19320.9)	849 (123.1)	0.64
Asthma – Yes	63 463	43 297 (68224)	12 984 (20459.2)	124 (195.4)	0.96
Blood cancer – No	752 348	463 376 (61590.6)	146 099 (19419.1)	964 (128.1)	0.66
Blood cancer – Yes	519	399 (76878.6)	84 (16185)	9 (1734.1)	10.71
Cerebral palsy – No	751 483	462 807 (61585.8)	146 013 (19430)	962 (128)	0.66
Cerebral palsy – Yes	1385	968 (69891.7)	170 (12274.4)	11 (794.2)	6.47
Chronic kidney disease – No	752 741	463 687 (61599.8)	146 157 (19416.6)	968 (128.6)	0.66
Chronic kidney disease – Yes	126	88 (69841.3)	26 (20634.9)	5 (3968.3)	19.23
Congenital heart disease – No	746 993	459 920 (61569.5)	145 137 (19429.5)	955 (127.8)	0.66
Congenital heart disease – Yes	5874	3855 (65628.2)	1046 (17807.3)	18 (306.4)	1.72
Diabetes type 1 – No	750 386	462 044 (61574.2)	145 704 (19417.2)	958 (127.7)	0.66
Diabetes type 1 – Yes	2481	1731 (69770.3)	479 (19306.7)	15 (604.6)	3.13
Diabetes type 2 – No	752 348	463 413 (61595.6)	146 100 (19419.2)	967 (128.5)	0.66
Diabetes type 2 – Yes	520	362 (69615.4)	83 (15961.5)	6 (1153.8)	7.23
Epilepsy – No	748 402	460 828 (61574.9)	145 474 (19437.9)	945 (126.3)	0.65
Epilepsy – Yes	4465	2947 (66002.2)	709 (15879.1)	28 (627.1)	3.95
Learning disability – No	722 557	446 300 (61766.8)	141 814 (19626.7)	910 (125.9)	0.64
Learning disability – Yes – not Downs syndrome	29 616	17 038 (57529.7)	4290 (14485.4)	58 (195.8)	1.35
Learning disability – Yes – Downs syndrome	694	437 (62968.3)	79 (11383.3)	5 (720.5)	6.33
Fracture – No	735 079	451 591 (61434.3)	142 030 (19321.7)	934 (127.1)	0.66
Fracture – Yes	17 789	12 184 (68491.8)	4153 (23345.9)	39 (219.2)	0.94
Rare pulmonary diseases – No	752 334	463 378 (61592.1)	146 100 (19419.6)	964 (128.1)	0.66
Rare pulmonary diseases – Yes	533	397 (74484.1)	83 (15572.2)	9 (1688.6)	10.84
Severe mental illness – No	751 404	462 817 (61593.6)	145 948 (19423.4)	967 (128.7)	0.66
Severe mental illness – Yes	1463	958 (65481.9)	235 (16062.9)	6 (410.1)	2.55
Sickle cell disease – No	752 468	463 514 (61599.2)	146 115 (19418.1)	958 (127.3)	0.66
Sickle cell disease – Yes	400	261 (65250)	68 (17000)	15 (3750)	22.06

10 907 had more than one risk group. Among those who have more than one risk group, 54 (495.1 per 100 000) had COVID-19 hospitalization. Learning disability refers to children with major intellectual disabilities or developmental challenges. Severe mental illness consists of bipolar affective disorder, psychosis, schizophrenia or schizoaffective disorder and severe depression.

The risk groups associated with an increased risk of COVID-19 hospital admission were: sickle cell disease (adjusted HR (95% CIs)) 14.35 (8.48-24.28), chronic kidney disease 11.34 (4.61-27.87), blood cancer 6.32 (3.24-12.35), rare pulmonary diseases 5.04 (2.58-9.86), type 2 diabetes 3.04 (1.34-6.92), epilepsy 2.54 (1.69-3.81), type 1 diabetes 2.48 (1.47-4.16), Down syndrome 2.45 (0.96-6.25), cerebral palsy 2.37 (1.26-4.47), severe mental illness 1.43 (0.63-3.24), fracture 1.41 (1.02-1.95), congenital heart disease 1.35 (0.82-2.23), asthma 1.28 (1.06-1.55) and learning disability (excluding Down syndrome) 1.08 (0.82-1.42), when compared to those without that condition (**Table 2**). Adjusted HRs were slightly higher in children aged 5-11 years old compared to those aged 12-17 years in all risk groups except congenital heart disease, type 1 diabetes and Down syndrome with overlapping 95% confidence intervals (Table S7 in the **Online Supplementary Document**). Similar results were observed when considering only admissions where SARS-CoV-2 was recoded as a reason for the admission and when admissions within 14 days were considered instead (**Table 2**).

**Table 2:** Hazard ratios for COVID-19 hospitalization, testing positive with SARS-CoV-2 and being tested comparing those with and without risk condition of interest among those 5-17 years old

Risk group			COVID-19 hospital admissions				Testing positive with SARS-CoV-2		Being tested	
	COVID-19 as reason for admission		Within 28 d of a positive test		Within 14 d of a positive test					
	Number of events	HR (95% CI)	Number of events	HR (95% CI)	Number of events	HR (95% CI)	Number of events	HR (95% CI)	Number of events	HR (95% CI)
Asthma	64	1.44 (1.1-1.88)	124	1.28 (1.06-1.55)	86	1.21 (0.97-1.53)	12 984	1.15 (1.13-1.17)	43 297	1.21 (1.2-1.22)
Blood cancer	7	9.82 (4.59-21)	9	6.32 (3.24-12.35)	9	8.56 (4.35-16.88)	84	1.14 (0.92-1.42)	399	1.62 (1.46-1.78)
Cerebral palsy	6	2.5 (1.05-5.94)	11	2.37 (1.26-4.47)	7	1.88 (0.84-4.2)	170	0.78 (0.67-0.91)	968	1.26 (1.18-1.34)
Chronic kidney disease	<5	7.64 (1.64-35.56)	5	11.34 (4.61-27.87)	5	14.39 (5.4-38.34)	26	1.28 (0.87-1.89)	88	1.21 (0.98-1.49)
Congenital heart disease	10	1.21 (0.55-2.64)	18	1.35 (0.82-2.23)	15	1.43 (0.79-2.6)	1046	1 (0.94-1.07)	3855	1.1 (1.06-1.13)
Diabetes type 1	5	1.79 (0.73-4.38)	15	2.48 (1.47-4.16)	13	3.05 (1.74-5.34)	479	1 (0.92-1.1)	1731	1.12 (1.07-1.18)
Diabetes type 2	<5	2.06 (0.49-8.65)	6	3.04 (1.34-6.92)	4	2.74 (0.99-7.57)	83	0.85 (0.68-1.05)	362	1.13 (1.02-1.25)
Epilepsy	15	2.74 (1.57-4.79)	28	2.54 (1.69-3.81)	24	3.17 (2.05-4.91)	709	0.9 (0.84-0.97)	2947	1.1 (1.06-1.14)
Learning disability – excluding Down syndrome	34	1.31 (0.91-1.89)	58	1.08 (0.82-1.42)	44	1.09 (0.8-1.49)	4290	0.71 (0.69-0.73)	17 038	0.91 (0.89-0.92)
Learning disability – Down syndrome	5	5.22 (1.83-14.87)	5	2.45 (0.96-6.25)	5	3 (1.11-8.14)	79	0.56 (0.45-0.71)	437	0.93 (0.84-1.02)
Fracture	18	1.42 (0.88-2.27)	39	1.41 (1.02-1.95)	27	1.36 (0.92-2)	4153	1.26 (1.22-1.3)	12 184	1.16 (1.14-1.18)
Rare pulmonary diseases	5	5.13 (2.11-12.45)	9	5.04 (2.58-9.86)	6	4.41 (1.96-9.92)	83	1.04 (0.84-1.29)	397	1.46 (1.32-1.61)
Severe mental illness	0	-	6	1.43 (0.63-3.24)	4	1.32 (0.49-3.56)	235	0.82 (0.72-0.93)	958	1.06 (0.99-1.13)
Sickle cell disease	14	28.49 (15.83-51.28)	15	14.35 (8.48-24.28)	15	19.76 (11.32-34.49)	68	0.96 (0.76-1.22)	261	1.1 (0.97-1.24)

HR – hazard ratio, CI – confidence interval

This table only included the subset of children with the selected conditions of interests who were admitted to hospitals with COVID-19 as the reason for admission. Reference group is children without the condition. Hazard ratios were derived using cox proportional hazard model adjusting for age, sex, socioeconomic status, other risk groups of interest, and prior hospitalization. Learning disability refers to children with major intellectual disabilities or developmental challenges.

Moreover, these risk groups were similarly associated with an increased hazard of being tested for SARS-CoV-2 (**Table 2**, Table S5 in the **Online Supplementary Document**), but were not necessarily associated with an increased hazard of SARS-CoV-2 infection (**Table 2**, Table S6 in the **Online Supplementary Document**). The rate of all-cause hospitalization among children in each category who did not have COVID-19 during the study period was higher than those without the specific risk group (Table S8 in the **Online Supplementary Document**). The unadjusted hazard ratios are shown in Table S10 and Figure S5 in the **Online Supplementary Document** and for each condition these are higher for hospital admission than the adjusted hazard ratios (**Table 2**), albeit with overlapping confidence intervals) and the full model of adjusted hazard ratios is available in Table S11 in the **Online Supplementary Document**.

The temporal trend of cumulative incidence of COVID-19 hospitalization between those with and without a risk condition since March 1, 2020 showed that the difference was highest during the third wave (May to July 2021) and lowest during the first wave (March to July 2020) (Figure S2 in the **Online Supplementary Document**). This was partly due to school closures during the first- and second-waves and different dominant variants. When stratified by time periods reflecting different dominant variants, the difference in cumulative incidence was higher when the Delta variant of concern (VOC) was dominating (May to July 2021) and lower when Alpha VOC was dominating (January to April 2021) (Figure S2 in the **Online Supplementary Document**). However, Table S12 in the **Online Supplementary Document** shows that the confidence intervals for the adjusted hazard ratios for COVID-19 hospitalization comparing those with and without a risk condition were wide for all three periods. There was no evidence of a greater impact of having a risk condition when Alpha or Delta VOCs were dominant.

## DISCUSSION

This national evaluation has found that, in children and young people aged 5-17 years old, selected underlying health conditions were associated with an increased risk of COVID-19 hospital admissions (although a number of these were not statistically significant). The proportion of hospital admissions among those who tested positive was consistently higher within each of these risk groups. This would translate into 66 415 (of 752 867, 8.8%) children aged 12-17 years old in Scotland with one of these underlying health conditions of interest. With the increased vaccine coverage over time, the risk of COVID-19 hospitalization would be anticipated to decrease. In considering vaccination policy, any potential benefits of vaccination in reducing the risk of SARS-CoV-2 infection and COVID-19 outcomes needs to be counterbalanced with an assessment of the risks of adverse events associated with vaccination.

This is, to our knowledge, the first national population-level study assessing the risk of SARS-CoV-2 infections and COVID-19 hospitalizations among school-aged children with underlying health conditions. Our study has several strengths. We developed a national scale data set platform and have enabled rapid link and access to and analysis of data from routinely collected electronic health record data and national databases in near real-time. Therefore, our study is less prone to recall bias and misclassification bias compared to studies using the samples of the population. The use of a large population aided study power, allowing estimation of hazard ratios in different risk groups. We are likely to have excellent generalisability across the UK and potentially across other countries with similar demographics and health systems.

Our study also has several limitations. Some cases may have been admitted for another reason. We did not have access to detailed case notes to validate the data linkage. As the definition of a COVID-19 hospital admission might not be stringent as we would have liked, we carried out two additional sensitivity analyses restricted to admissions where SARS-CoV-2 was listed as one of the reasons for hospital admission and when admissions within 14 days were considered instead, and the hazard ratios were similar as for the more general definition (**Table 2**). The number of intensive care unit admissions and deaths were very small in this population (nine overall in spite of the fact that the definition of a COVID-19 related deaths might not be specific enough), so we were unable to evaluate these more severe outcomes. The number of events observed was small which might affect the accuracy of the estimates. We only included risk groups that were defined by the QCOVID prediction algorithm [15] (this was based on adult population) and that had at least five events of hospitalizations during the study period, so we may have missed some important pediatric risk groups (eg, children with chronic severe lung disease, children with complex medical conditions including home ventilated patients). School-aged children with underlying health conditions had an increased rate of being tested, which was possibly because they were more likely to require hospital admission for non-COVID-19 reasons and were therefore subjected to repeated SARS-CoV-2 testing and screening in hospital than those in low-risk groups. But our data show that they did not have an increased rate of testing positive with SARS-CoV-2. One theory for increased risk in these children is their risk of exposure and infection as their care and support needs mean that they require contact with more people. This did not seem to be the case from this analysis; rather the risk was of higher rates of hospitalization for a given level of infection, which may introduce some potential ascertainment bias. It is possible that certain risk group actually were less vulnerable to serious manifestations of SARS-CoV-2 infection. There may also have been different health care seeking behaviors and lower threshold for COVID-19 admission in children with an underlying health condition, which may have resulted in higher chances of being tested for SARS-CoV-2. In addition, the procedure/schedule for RT-PCR testing among the cohort varied over the pandemic – for example, prior to lateral flow tests being available, RT-PCR testing was just for symptomatic cases. After the introduction of lateral flow tests, people would also get a follow up RT-PCR testing if they were positive on lateral flow tests. Although our Cox models were adjusted for a number of potential confounders, we were unable to adjust for some risk factors (eg, obesity/BMI and ethnicity which could also potentially operate as effect modifiers) due to lack of reliable recording in Scottish electronic health records raising the possibility of residual confounding. We did not look at myocarditis and long-COVID. We were also unable to investigate Paediatric Multisystem Inflammatory Syndrome temporally associated with SARS-CoV-2 (PIMS-TS) because these syndromes were only routinely recorded in the Scotland national data sets from January 1, 2021 onwards; this outcome was not captured across all of our study period and was therefore not included in our analysis [18]. The majority of cases of PIMS-TS resulted in intensive care unit (ICU) admissions and given the very low number of pediatric admissions for COVID-19 across Scotland during the study period this is unlikely to have had any major impact on our findings [19]. In a cross-sectional study of a large cohort of patients with Multisystem Inflammatory Syndrome in Children (MIS-C) from the US, 63.3% of school-aged children (5-17 years old) with MIS-C were admitted to ICU [20]. We need to take all these limitations as well as, more fundamentally, inherent challenges with respect to the nature and reliability of hospital-based data into consideration when using them to inform any policy-making.

Similar results have been reported in other studies [7-9,21-31]. A systematic review containing over 9000 children with comorbidities and over 275 000 children without comorbidities found that children with comorbidities had a higher risk of severe COVID-19 (relative risk ratio (RR) = 1.79 (95% CI = 1.27-2.51)) and associated mortality (RR = 2.81 (1.31-6.02)) than children without underlying comorbidities [8]. Similar findings were reported in another systematic review indicating that the majority of children who required ventilation for SARS-CoV-2 infection had underlying comorbidities [9]. In one study of over 27 000 US children with confirmed COVID-19, African American (odds ratio (OR) = 2.28 (95% CI = 1.93-2.70)) or mixed race (OR = 2.95 (2.28-3.82)) and an underlying medical condition (OR = 3.55 (3.14-4.01)) were found to be strong predictors of hospitalization [7]. Also, children with a prior medical condition had an increased risk of COVID-19 death (OR = 8.80 (3.70-21.10)). Our study has added more robust and generalizable evidence than previous studies using standard definitions of underlying conditions and identifying outcomes in routinely collected clinical records at national population-level and quantified the strength of associations between each risk group of interest and COVID-19 hospitalization.

Building on this work, it is important for more detailed characterization of different risk groups for severe COVID-19 outcomes in children and to investigate underlying mechanisms that predispose such children to these increased risks. With vaccines in school-aged children being given and planned nationally and internationally, policymakers will be able to use our study’s data and findings to inform decisions on vaccination priorities among school-aged children, together with other public health surveillance data.

## CONCLUSIONS

In summary, we provide national evidence that children and young people aged 5-17 years old with select underlying health conditions were associated with an increased risk of COVID-19 hospital admissions in Scotland. The findings from this linkage of multiple data sources can help inform the prioritization of school-aged children for vaccines based on having one of these conditions.

## Additional material


Online Supplementary Document
