# Supplementary material for: Risk of COVID-19 hospitalizations among school-aged children in Scotland: A national incident cohort study
Source: J Glob Health. 2022 Sep 23;12:05044. doi: 10.7189/jogh.12.05044 (PMC9494196; doi:10.7189/jogh.12.05044)
Supplement: Online Supplementary Document [file jogh-12-05044-s001.pdf]

ONLINE SUPPLEMENTARY DOCUMENT

Title: Risk of COVID-19 hospitalizations among school-aged children in Scotland: a national incident cohort study

Authors: Ting Shi*, Jiafeng Pan*, Emily Moore, Srinivasa Vittal Katikireddi, Annemarie B Docherty, Lynda Fenton, Colin McCowan, Utkarsh Agrawal, Steven Kerr, Syed Ahmar Shah, Sarah J Stock, Colin R Simpson, Chris Robertson, Aziz Sheikh, on behalf of Public Health Scotland and the EAVE II Collaborators

* Joint first authors contributed equally

The online supplementary document (OSD) has a free format.
Submit the OSD as PDF.
If you have complex accompanying data or information that does not translate well into the PDF format (eg, datasheets or tools), consider using registers for such data, such as OSF or Zotero.
In the manuscript text, provide the link to the registered data.

Figures in the Online Supplementary Document should be numbered in this way: S1, S2, S3, etc., and cited in the text. Example: (Table S1 in the **Online Supplementary Document**).

S1 Box: Predictor variables in the QCOVID algorithm

- accommodation (homeless, care home, neither)
- asthma
- atrial fibrillation
- blood cancer
- body mass index (BMI)
- cerebral palsy
- chronic kidney disease
- cirrhosis of liver
- congenital heart disease
- congestive cardiac failure
- chronic obstructive pulmonary disease (COPD)
- coronary heart disease
- dementia
- diabetes 1
- diabetes 2
- epilepsy
- ethnicity
- learning disability
- fracture
- Parkinson's disease
- peripheral vascular disease
- pulmonary hypertension or pulmonary fibrosis
- rare neurological conditions
- rare pulmonary diseases
- respiratory cancer
- rheumatoid arthritis or systemic lupus erythematosus
- severe mental illness
- sickle cell disease
- stroke
- venous thromboembolism

Table S1: ICD-10 codes for COVID-19 illness

Code	Description
U07.1	COVID-19, virus identified
U07.2	COVID-19, virus not identified
Source: https://www.who.int/classifications/icd/COVID-19-coding-icd10.pdf	

ICD-10: International Classification of Diseases 10.

Table S2: List of risk groups of interest included in this study (using a cut-off of at least 5 events of COVID-19 hospitalizations)

Risk group of interest*	Coding
Asthma	Yes/no
Blood cancer	Yes/no
Cerebral palsy	Yes/no
Chronic kidney disease	Yes/no
Congenital heart disease	Yes/no
Diabetes type 1	Yes/no
Diabetes type 2	Yes/no
Epilepsy	Yes/no
Learning disability	Yes without Down’s syndrome/yes with Down’s syndrome/no
Fracture	Yes/no
Rare pulmonary diseases	Yes/no
Severe mental illness	Yes/no
Sickle cell disease	Yes/no

*Body mass index data were missing on 93.1% and ethnicity data were missing on 31.1% of participants; these variables were therefore not included.

Table S3: Reporting STROBE checklist for cohort studies

	Item No	Recommendation	Location
**Title and abstract**	1	(*a*) Indicate the study's design with a commonly used term in the title or the abstract	Title and Abstract (Methods and Findings)
		(*b*) Provide in the abstract an informative and balanced summary of what was done and what was found	Abstract (Methods and Findings)
**Introduction**			
Background/rationale	2	Explain the scientific background and rationale for the investigation being reported	Introduction paragraphs 1-3
Objectives	3	State specific objectives, including any prespecified hypotheses	Introduction paragraph 4
**Methods**			
Study design	4	Present key elements of study design early in the paper	Methods (Study design) paragraph 2
Setting	5	Describe the setting, locations, and relevant dates, including periods of recruitment, exposure, follow-up, and data collection	Methods (Study design) paragraph 2 and Methods (Statistical analysis) paragraph 1
Participants	6	(*a*) Give the eligibility criteria, and the sources and methods of selection of participants. Describe methods of follow-up	Methods (Study design) paragraph 2 and Methods (Data sources)
		(*b*) For matched studies, give matching criteria and number of exposed and unexposed	NA
Variables	7	Clearly define all outcomes, exposures, predictors, potential confounders, and effect modifiers. Give diagnostic criteria, if applicable	Methods (Outcomes and Risk groups)
Data sources/ measurement	8*	For each variable of interest, give sources of data and details of methods of assessment (measurement). Describe comparability of assessment methods if there is more than one group	Methods (Data sources)
Bias	9	Describe any efforts to address potential sources of bias	Methods (Statistical analysis) paragraph 2
Study size	10	Explain how the study size was arrived at	Methods (Study design) paragraph 2
Quantitative variables	11	Explain how quantitative variables were handled in the analyses. If applicable, describe which groupings were chosen and why	Methods (Statistical analysis) paragraph 2
Statistical methods	12	(*a*) Describe all statistical methods, including those used to control for confounding	Methods (Statistical analysis) paragraphs 2-3
		(*b*) Describe any methods used to examine subgroups and interactions	Methods (Statistical analysis) paragraph 2
		(*c*) Explain how missing data were addressed	Methods (Risk groups)
		(*d*) If applicable, explain how loss to follow-up was addressed	NA
		(*e*) Describe any sensitivity analyses	NA
**Results**			
Participants	13*	(a) Report numbers of individuals at each stage of study—eg numbers potentially eligible, examined for eligibility, confirmed eligible,	Results paragraph 1

		included in the study, completing follow-up, and analysed	
		(b) Give reasons for non-participation at each stage	NA
		(c) Consider use of a flow diagram	Methods (Data sources)
Descriptive data	14*	(a) Give characteristics of study participants (eg demographic, clinical, social) and information on exposures and potential confounders	Results paragraphs 1-2
		(b) Indicate number of participants with missing data for each variable of interest	Methods (Risk groups), Table S2
		(c) Summarise follow-up time (eg, average and total amount)	Results paragraph 1
Outcome data	15*	Report numbers of outcome events or summary measures over time	Results paragraph 1
Main results	16	(*a*) Give unadjusted estimates and, if applicable, confounder-adjusted estimates and their precision (eg, 95% confidence interval). Make clear which confounders were adjusted for and why they were included	Results paragraphs 2-4
		(*b*) Report category boundaries when continuous variables were categorized	NA
		(*c*) If relevant, consider translating estimates of relative risk into absolute risk for a meaningful time period	NA
Other analyses	17	Report other analyses done—eg analyses of subgroups and interactions, and sensitivity analyses	Results paragraph 4&5
**Discussion**			
Key results	18	Summarise key results with reference to study objectives	Discussion paragraph 1
Limitations	19	Discuss limitations of the study, taking into account sources of potential bias or imprecision. Discuss both direction and magnitude of any potential bias	Discussion paragraph 1
Interpretation	20	Give a cautious overall interpretation of results considering objectives, limitations, multiplicity of analyses, results from similar studies, and other relevant evidence	Discussion paragraphs 1, 5 and 6
Generalisability	21	Discuss the generalisability (external validity) of the study results	Discussion paragraphs 6
**Other information**			
Funding	22	Give the source of funding and the role of the funders for the present study and, if applicable, for the original study on which the present article is based	Submission form

*Give information separately for exposed and unexposed groups. STROBE: Strengthening the Reporting of Observational studies in Epidemiology. NA: not applicable.

Table S4: Baseline characteristics by each risk group of interest

Variable	Level	Overall	Asthma	Blood cancer	Cerebral palsy	Chronic kidney disease	Congenital heart disease	Diabetes type 1	Diabetes type 2	Epilepsy	Learning disability $	Down's syndrome	Fracture	Rare pulmonary diseases	Severe mental illness	Sickle cell disease
Total		752867 (100)	63463 (100)	519 (100)	1385 (100)	126 (100)	5874 (100)	2481 (100)	520 (100)	4465 (100)	29616 (100)	694 (100)	17789 (100)	533 (100)	1463 (100)	400 (100)
Age	5-11 years old	416713 (55.4)	25967 (40.9)	239 (46)	702 (50.7)	56 (44.3)	3150 (53.6)	958 (38.6)	173 (33.3)	1976 (44.3)	14581 (49.2)	409 (59)	6466 (36.4)	265 (49.6)	94 (6.4)	214 (53.6)
Age	12-17 years old	336154 (44.6)	37495 (59.1)	280 (54)	683 (49.3)	70 (55.7)	2724 (46.4)	1523 (61.4)	346 (66.7)	2489 (55.7)	15036 (50.8)	285 (41)	11322 (63.6)	269 (50.4)	1369 (93.6)	185 (46.4)
Age	Median (IQR)	11(8,14)	13(10,15)	12(9,15)	11(9,14)	13(9,15)	11(8,14)	13(10,15)	13(10,16)	12(9,15)	12(9,15)	11(8,14)	13(10,15)	12(9,14)	16(15,17)	11(9,14.75)
Sex	Female	367680 (48.8)	26364 (41.5)	225 (43.4)	578 (41.8)	44 (35.1)	2922 (49.7)	1185 (47.7)	268 (51.7)	2062 (46.2)	8015 (27.1)	333 (47.9)	7881 (44.3)	256 (48)	907 (62)	185 (46.3)
Sex	Male	385187 (51.2)	37099 (58.5)	294 (56.6)	806 (58.2)	82 (64.9)	2952 (50.3)	1297 (52.3)	251 (48.3)	2403 (53.8)	21602 (72.9)	362 (52.1)	9908 (55.7)	277 (52)	556 (38)	215 (53.7)
SIMD*	1 - High	167049 (22.2)	15908 (25.1)	98 (18.9)	344 (24.9)	29 (23.4)	1322 (22.5)	476 (19.2)	119 (22.9)	1153 (25.8)	8592 (29)	152 (21.9)	4348 (24.4)	130 (24.4)	285 (19.5)	106 (26.6)
SIMD	2	146036 (19.4)	13265 (20.9)	97 (18.8)	307 (22.2)	28 (22.6)	1101 (18.7)	488 (19.7)	125 (24.1)	903 (20.2)	6664 (22.5)	126 (18.2)	3380 (19)	104 (19.5)	307 (21)	84 (20.9)
SIMD	3	135285 (18)	11595 (18.3)	103 (19.9)	257 (18.5)	23 (18)	1109 (18.9)	472 (19)	98 (18.8)	802 (18)	5211 (17.6)	137 (19.7)	2960 (16.6)	98 (18.5)	312 (21.3)	73 (18.2)
SIMD	4	147272 (19.6)	11566 (18.2)	120 (23.1)	253 (18.3)	28 (21.9)	1158 (19.7)	553 (22.3)	95 (18.3)	782 (17.5)	4947 (16.7)	145 (20.9)	3313 (18.6)	100 (18.8)	320 (21.9)	67 (16.8)
SIMD	5 - Low	150054 (19.9)	10617 (16.7)	96 (18.5)	211 (15.2)	16 (12.5)	1144 (19.5)	475 (19.2)	76 (14.7)	786 (17.6)	3982 (13.4)	131 (18.9)	3646 (20.5)	97 (18.3)	218 (14.9)	68 (17)
SIMD	NA	7172 (1)	512 (0.8)	<5	13 (0.9)	<5	40 (0.7)	18 (0.7)	6 (1.1)	39 (0.9)	221 (0.7)	<5	141 (0.8)	<5	21 (1.5)	<5
Number of hospitalizations#	0	674384 (89.6)	53580 (84.4)	265 (51.2)	699 (50.5)	68 (53.8)	4513 (76.8)	1352 (54.5)	245 (47.2)	2779 (62.2)	24301 (82.1)	426 (61.4)	15171 (85.3)	235 (44.1)	1085 (74.2)	228 (57.1)
Number of hospitalizations	1+	78483 (10.4)	9883 (15.6)	253 (48.8)	685 (49.5)	58 (46.2)	1361 (23.2)	1130 (45.5)	274 (52.8)	1686 (37.8)	5315 (17.9)	268 (38.6)	2618 (14.7)	298 (55.9)	378 (25.8)	171 (42.9)

Data were presented as n (%) or median (IQR). *1 indicates most deprived, 5 indicates least deprived. $learning disability excluding Down's syndrome. SIMD: Scottish Index of Multiple Deprivation. #Number of hospitalizations in the two years prior to March 1, 2020.

Table S5: Hazard ratios for being tested comparing those with and without risk condition of interest in each age group (5-17, 5-11, 12-17 years)

Risk group	5 to 17 years	5 to 11 years	12 to 17 years
	HR (95% CI)	HR (95% CI)	HR (95% CI)
Asthma	1.21 (1.2-1.22)	1.26 (1.25-1.28)	1.17 (1.16-1.19)
Blood cancer	1.62 (1.46-1.78)	1.88 (1.63-2.16)	1.43 (1.25-1.65)
Cerebral palsy	1.26 (1.18-1.34)	1.45 (1.33-1.58)	1.09 (1-1.2)
Chronic kidney disease	1.21 (0.98-1.49)	1.36 (0.99-1.87)	1.16 (0.87-1.54)
Congenital heart disease	1.1 (1.06-1.13)	1.11 (1.06-1.16)	1.08 (1.03-1.14)
Diabetes type 1	1.12 (1.07-1.18)	1.16 (1.07-1.25)	1.1 (1.03-1.17)
Diabetes type 2	1.13 (1.02-1.25)	1.11 (0.92-1.33)	1.13 (1-1.28)
Epilepsy	1.1 (1.06-1.14)	1.17 (1.11-1.23)	1.06 (1.01-1.11)
Learning disability - excluding Down's syndrome	0.91 (0.89-0.92)	1.02 (0.99-1.04)	0.82 (0.8-0.83)
Learning disability - Down's syndrome	0.93 (0.84-1.02)	1.01 (0.89-1.14)	0.83 (0.71-0.96)
Fracture	1.16 (1.14-1.18)	1.16 (1.13-1.2)	1.16 (1.14-1.19)
Rare pulmonary diseases	1.46 (1.32-1.61)	1.47 (1.28-1.69)	1.45 (1.26-1.66)
Severe mental illness	1.06 (0.99-1.13)	0.77 (0.58-1.01)	1.07 (1-1.15)
Sickle cell disease	1.1 (0.97-1.24)	1.11 (0.94-1.32)	1.08 (0.9-1.3)

Reference group is children without the condition. HR: Hazard Ratio. CI: Confidence Interval. Hazard ratios were derived using Cox proportional hazard model adjusting for age, sex, socioeconomic status, other risk groups of interest, and prior hospitalization.

Table S6: Hazard ratios for testing positive with SARS-CoV-2 comparing those with and without risk condition of interest in each age group (5-17, 5-11, 12-17 years)

Risk group	5 to 17 years	5 to 11 years	12 to 17 years
	HR (95% CI)	HR (95% CI)	HR (95% CI)
Asthma	1.15 (1.13-1.17)	1.22 (1.19-1.25)	1.1 (1.08-1.13)
Blood cancer	1.14 (0.92-1.42)	1.39 (1.02-1.89)	0.99 (0.73-1.33)
Cerebral palsy	0.78 (0.67-0.91)	0.96 (0.79-1.18)	0.64 (0.51-0.81)
Chronic kidney disease	1.28 (0.87-1.89)	1.68 (0.97-2.9)	1.09 (0.63-1.89)
Congenital heart disease	1 (0.94-1.07)	1.07 (0.99-1.17)	0.93 (0.84-1.02)
Diabetes type 1	1 (0.92-1.1)	1.17 (1.02-1.34)	0.91 (0.8-1.02)
Diabetes type 2	0.85 (0.68-1.05)	0.9 (0.62-1.31)	0.81 (0.62-1.06)
Epilepsy	0.9 (0.84-0.97)	1.05 (0.94-1.17)	0.81 (0.73-0.9)
Learning disability - excluding Down’s syndrome	0.71 (0.69-0.73)	0.84 (0.81-0.88)	0.61 (0.58-0.64)
Learning disability - Down’s syndrome	0.56 (0.45-0.71)	0.67 (0.51-0.89)	0.44 (0.3-0.64)
Fracture	1.26 (1.22-1.3)	1.24 (1.18-1.31)	1.27 (1.22-1.32)
Rare pulmonary diseases	1.04 (0.84-1.29)	1.04 (0.76-1.43)	1.04 (0.77-1.41)
Severe mental illness	0.82 (0.72-0.93)	0.64 (0.37-1.1)	0.83 (0.72-0.95)
Sickle cell disease	0.96 (0.76-1.22)	1.1 (0.8-1.5)	0.83 (0.57-1.21)

Reference group is children without the condition. * Number of testing positive with SARS-CoV-2 among 5-17 years old. HR: Hazard Ratio. CI: Confidence Interval. Hazard ratios were derived using Cox proportional hazard model adjusting for age, sex, socioeconomic status, other risk groups of interest, and prior hospitalization.

Table S7: Hazard ratios for COVID-19 hospitalization comparing those with and without risk condition of interest in each age group (5-17, 5-11, 12-17 years)

Risk group	5 to 17 years	5 to 11 years	12 to 17 years
	HR (95% CI)	HR (95% CI)	HR (95% CI)
Asthma	1.28 (1.06-1.55)	1.67 (1.24-2.26)	1.1 (0.86-1.42)
Blood cancer	6.32 (3.24-12.35)	16.17 (7.77-33.66)	1.08 (0.15-8.08)
Cerebral palsy	2.37 (1.26-4.47)	3.13 (1.38-7.1)	1.64 (0.58-4.6)
Chronic kidney disease	11.34 (4.61-27.87)	18.4 (5.68-59.68)	7.39 (1.77-30.77)
Congenital heart disease	1.35 (0.82-2.23)	1.31 (0.62-2.76)	1.35 (0.69-2.67)
Diabetes type 1	2.48 (1.47-4.16)	2.78 (1.14-6.78)	2.3 (1.21-4.36)
Diabetes type 2	3.04 (1.34-6.92)	NA	4.02 (1.76-9.18)
Epilepsy	2.54 (1.69-3.81)	3.2 (1.77-5.76)	2.2 (1.25-3.88)
Learning disability - excluding Down's syndrome	1.08 (0.82-1.42)	1.4 (0.96-2.06)	0.86 (0.58-1.29)
Learning disability - Down's syndrome	2.45 (0.96-6.25)	1.93 (0.44-8.41)	3.05 (0.89-10.44)
Fracture	1.41 (1.02-1.95)	1.45 (0.8-2.65)	1.41 (0.96-2.08)
Rare pulmonary diseases	5.04 (2.58-9.86)	6.16 (2.24-16.91)	4.24 (1.71-10.48)
Severe mental illness	1.43 (0.63-3.24)	NA	1.42 (0.63-3.23)
Sickle cell disease	14.35 (8.48-24.28)	20.94 (10.53-41.65)	9.92 (4.32-22.81)

Reference group is children without the condition. COVID-19 hospitalization is within 28 days of a positive test. HR: Hazard Ratio. CI: Confidence Interval. NA: not included in the model due to zero event of COVID-19 hospitalization. Hazard ratios were derived using cox proportional hazard model adjusting for age, sex, socioeconomic status, other risk groups of interest, and prior hospitalization.

Table S8 : Number and rate of all cause hospitalization in each risk group of interest among children and young people aged 5 to 17 years old who did not test positive for COVID-19 from March 1, 2020 to November 22, 2021

Risk group	Number without COVID-19	Number with hospitalization among those without COVID-19	Rate of hospitalization per 100 of those without COVID-19
Asthma - No	556205	31394	5.6
Asthma - Yes	50479	4448	8.8
Blood cancer - No	606249	35678	5.9
Blood cancer - Yes	435	164	37.7
Cerebral palsy - No	605470	35398	5.9
Cerebral palsy - Yes	1215	444	36.5
Chronic kidney disease - No	606584	35809	5.9
Chronic kidney disease - Yes	100	33	33.0
Congenital heart disease - No	601856	35236	5.9
Congenital heart disease - Yes	4828	606	12.6
Diabetes type 1 - No	604682	35342	5.8
Diabetes type 1 - Yes	2002	500	25.0
Diabetes type 2 - No	606248	35697	5.9
Diabetes type 2 - Yes	437	145	33.2
Epilepsy - No	602928	34971	5.8
Epilepsy - Yes	3756	871	23.2
Learning disability - No	580743	33054	5.7
Learning disability - Yes - not Downs syndrome	25326	2657	10.5
Learning disability - Yes - Downs syndrome	615	131	21.3
Fracture - No	593049	34713	5.9
Fracture - Yes	13636	1129	8.3
Rare pulmonary diseases - No	606234	35681	5.9
Rare pulmonary diseases - Yes	450	161	35.8
Severe mental illness - No	605456	35582	5.9
Severe mental illness - Yes	1228	260	21.2
Sickle cell disease - No	606353	35750	5.9
Sickle cell disease - Yes	332	92	27.7

Table S9: Number of COVID-19 hospitalization stratified by testing positivity before and after admission for each risk group of interest among children and young people aged 5 to 17 years old

Risk group	Number of 5-17 years old	Number with COVID-19 hospitalization	Number testing positive on admission or while hospitalized	Number with positive test within 28 days before hospitalization /with COVID 19 as admission diagnosis code
Asthma - No	689404	849(123.1)	296(42.9)	553(80.2)
Asthma - Yes	63463	124(195.4)	37(58.3)	87(137.1)
Blood cancer - No	752348	964(128.1)	327(43.5)	637(84.7)
Blood cancer - Yes	519	9(1734.1)	NA	<5
Cerebral palsy - No	751483	962(128)	329(43.8)	633(84.2)
Cerebral palsy - Yes	1385	11(794.2)	<5	NA
Chronic kidney disease - No	752741	968(128.6)	329(43.7)	639(84.9)
Chronic kidney disease - Yes	126	5(3968.3)	<5	<5
Congenital heart disease - No	746993	955(127.8)	326(43.6)	629(84.2)
Congenital heart disease - Yes	5874	18(306.4)	7(119.2)	11(187.3)
Diabetes type 1 - No	750386	958(127.7)	326(43.4)	632(84.2)
Diabetes type 1 - Yes	2481	15(604.6)	7(282.1)	8(322.5)
Diabetes type 2 - No	752348	967(128.5)	329(43.7)	638(84.8)
Diabetes type 2 - Yes	520	6(1153.8)	<5	<5
Epilepsy - No	748402	945(126.3)	322(43)	623(83.2)
Epilepsy - Yes	4465	28(627.1)	11(246.4)	17(380.7)
Learning disability - No	722557	910(125.9)	316(43.7)	594(82.2)
Learning disability - Yes - not Downs syndrome	29616	58(195.8)	15(50.6)	43(145.2)
Learning disability - Yes - Downs syndrome	694	5(720.5)	<5	<5
Fracture - No	735079	934(127.1)	316(43)	618(84.1)
Fracture - Yes	17789	39(219.2)	17(95.6)	22(123.7)
Rare pulmonary diseases - No	752334	964(128.1)	328(43.6)	636(84.5)
Rare pulmonary diseases - Yes	533	9(1688.6)	NA	<5
Severe mental illness - No	751404	967(128.7)	330(43.9)	637(84.8)
Severe mental illness - Yes	1463	6(410.1)	<5	<5
Sickle cell disease - No	752468	958(127.3)	322(42.8)	636(84.5)

Sickle cell disease - Yes	400	15(3750)	NA	<5

NA: Not Available. Table was presented in n or n(%). The denominators for the % was the number of 5-17 years old.

Table S10: Univariable hazard ratios for being tested, testing positive with SARS-CoV-2 and COVID-19 hospitalization comparing 5-17 years old with and without risk condition of interest as well as the covariates included in the adjusted model

Risk group	Category	COVID-19 hospitalization	Testing positive with SARS-CoV-2	Being tested
		HR (95% CI)	HR (95% CI)	HR (95% CI)
Asthma	Yes vs. No	1.59 (1.31-1.92)	1.19 (1.17-1.21)	1.23 (1.21-1.24)
Blood cancer	Yes vs. No	13.7 (7.07-26.53)	1.24 (1-1.53)	1.77 (1.6-1.95)
Cerebral palsy	Yes vs. No	6.3 (3.47-11.44)	0.79 (0.68-0.92)	1.38 (1.29-1.47)
Chronic kidney disease	Yes vs. No	31.26 (12.87-75.94)	1.28 (0.87-1.88)	1.34 (1.09-1.66)
Congenital heart disease	Yes vs. No	2.39 (1.49-3.83)	0.99 (0.93-1.05)	1.13 (1.1-1.17)
Diabetes type 1	Yes vs. No	4.74 (2.83-7.93)	1.12 (1.02-1.23)	1.24 (1.18-1.3)
Diabetes type 2	Yes vs. No	8.92 (3.95-20.12)	0.96 (0.77-1.19)	1.28 (1.15-1.42)
Epilepsy	Yes vs. No	5 (3.43-7.3)	0.91 (0.84-0.98)	1.18 (1.13-1.22)
Learning disability - excluding Down's syndrome	Yes vs. No learning disability	1.56 (1.19-2.04)	0.72 (0.7-0.74)	0.92 (0.91-0.94)
Learning disability - Down's syndrome	Yes vs. No learning disability	5.68 (2.33-13.85)	0.59 (0.47-0.74)	1.05 (0.95-1.15)
Fracture	Yes vs. No	1.72 (1.25-2.38)	1.32 (1.28-1.37)	1.19 (1.17-1.21)
Rare pulmonary diseases	Yes vs. No	13.27 (6.85-25.73)	1.14 (0.92-1.41)	1.64 (1.49-1.81)
Severe mental illness	Yes vs. No	3.16 (1.4-7.14)	0.92 (0.81-1.05)	1.17 (1.09-1.24)
Sickle cell disease	Yes vs. No	30.24 (18.11-50.5)	1 (0.79-1.27)	1.19 (1.05-1.34)
Sex	M vs. F	0.79 (0.7-0.9)	0.91 (0.9-0.92)	0.95 (0.95-0.96)
SIMD*	1 - most deprived	1	1	1
	2	0.75 (0.62-0.9)	1.04 (1.02-1.05)	1.05 (1.04-1.06)
	3	0.63 (0.51-0.76)	0.93 (0.91-0.94)	1 (0.99-1.01)
	4	0.72 (0.6-0.87)	0.97 (0.96-0.99)	1.04 (1.03-1.05)
	5 - least deprived	0.5 (0.41-0.62)	1.1 (1.09-1.12)	1.14 (1.13-1.15)
	unknown	0.55 (0.26-1.17)	0.71 (0.67-0.76)	0.86 (0.84-0.89)
Number of hospitalizations$	0	1	1	1
	1+	4.04 (3.53-4.63)	1.21 (1.19-1.23)	1.29 (1.27-1.3)

Reference group is children without the condition. COVID-19 hospitalization is within 28 days of a positive test. HR: Hazard Ratio. CI: Confidence Interval. *1 indicates most deprived, 5 indicates least deprived. SIMD: Scottish Index of Multiple Deprivation. $Number of hospitalizations in the two years prior to March 1, 2020.

Table S11: Adjusted hazard ratios for being tested, testing positive with SARS-CoV-2 and COVID-19 hospitalization comparing 5-17 years old with and without risk condition of interest

Risk group	Category	COVID-19 hospitalization	Testing positive with SARS-CoV-2	Being tested
		HR (95% CI)	HR (95% CI)	HR (95% CI)
Asthma	Yes vs. No	1.28 (1.06-1.55)	1.15 (1.13-1.17)	1.21 (1.2-1.22)
Blood cancer	Yes vs. No	6.32 (3.24-12.35)	1.14 (0.92-1.42)	1.62 (1.46-1.78)
Cerebral palsy	Yes vs. No	2.37 (1.26-4.47)	0.78 (0.67-0.91)	1.26 (1.18-1.34)
Chronic kidney disease	Yes vs. No	11.34 (4.61-27.87)	1.28 (0.87-1.89)	1.21 (0.98-1.49)
Congenital heart disease	Yes vs. No	1.35 (0.82-2.23)	1 (0.94-1.07)	1.1 (1.06-1.13)
Diabetes type 1	Yes vs. No	2.48 (1.47-4.16)	1 (0.92-1.1)	1.12 (1.07-1.18)
Diabetes type 2	Yes vs. No	3.04 (1.34-6.92)	0.85 (0.68-1.05)	1.13 (1.02-1.25)
Epilepsy	Yes vs. No	2.54 (1.69-3.81)	0.9 (0.84-0.97)	1.1 (1.06-1.14)
Learning disability - excluding Down's syndrome	Yes vs. No learning disability	1.08 (0.82-1.42)	0.71 (0.69-0.73)	0.91 (0.89-0.92)
Learning disability - Down's syndrome	Yes vs. No learning disability	2.45 (0.96-6.25)	0.56 (0.45-0.71)	0.93 (0.84-1.02)
Fracture	Yes vs. No	1.41 (1.02-1.95)	1.26 (1.22-1.3)	1.16 (1.14-1.18)
Rare pulmonary diseases	Yes vs. No	5.04 (2.58-9.86)	1.04 (0.84-1.29)	1.46 (1.32-1.61)
Severe mental illness	Yes vs. No	1.43 (0.63-3.24)	0.82 (0.72-0.93)	1.06 (0.99-1.13)
Sickle cell disease	Yes vs. No	14.35 (8.48-24.28)	0.96 (0.76-1.22)	1.1 (0.97-1.24)
Sex	M vs. F	0.77 (0.68-0.88)	0.91 (0.9-0.92)	0.95 (0.94-0.95)
SIMD*	1 - most deprived	1	1	
	2	0.76 (0.63-0.91)	1.04 (1.02-1.05)	1.05 (1.04-1.06)
	3	0.64 (0.53-0.78)	0.93 (0.91-0.94)	1.01 (1-1.02)
	4	0.77 (0.64-0.92)	0.97 (0.96-0.99)	1.05 (1.04-1.06)
	5 - least deprived	0.54 (0.44-0.67)	1.1 (1.09-1.12)	1.15 (1.14-1.16)
	unknown	0.57 (0.27-1.21)	0.7 (0.66-0.75)	0.87 (0.84-0.9)
Number of hospitalizations$	0	1	1	
	1+	3.5 (3.03-4.03)	1.25 (1.22-1.27)	1.28 (1.26-1.29)

Reference group is children without the condition. COVID-19 hospitalization is within 28 days of a positive test. HR: Hazard Ratio. CI: Confidence Interval. Spline of age included in the model was shown in S4 Fig. *1 indicates most deprived, 5 indicates least deprived. SIMD: Scottish Index of Multiple Deprivation. $Number of hospitalizations in the two years prior to March 1, 2020.

Table S12: Adjusted hazard ratios for COVID-19 hospitalization comparing 5-17 years old with and without risk condition of interest – stratified by the period with different VOC

Risk group	Full study period		Period with wild type	Period with Alpha VOC dominant	Period with Delta VOC dominant
	Number of events	HR (95% CI)	HR (95% CI)	HR (95% CI)	HR (95% CI)
Asthma	124	1.28 (1.06-1.55)	1.43 (0.91-2.24)	2.03 (1.29-3.2)	1.13 (0.89-1.44)
Blood cancer	9	6.32 (3.24-12.35)	4.06 (0.55-29.81)	19.68 (6.05-64)	4.92 (2.01-12.06)
Cerebral palsy	11	2.37 (1.26-4.47)	3.31 (1.11-9.88)	3.63 (0.81-16.34)	1.83 (0.73-4.58)
Chronic kidney disease	5	11.34 (4.61-27.87)	NA	19.21 (2.54-145.33)	13.98 (5.1-38.36)
Congenital heart disease	18	1.35 (0.82-2.23)	1.95 (0.75-5.12)	2.23 (0.69-7.21)	1.05 (0.54-2.05)
Diabetes type 1	15	2.48 (1.47-4.16)	1.82 (0.45-7.4)	3.94 (1.21-12.75)	2.43 (1.28-4.59)
Diabetes type 2	6	3.04 (1.34-6.92)	NA	NA	4.66 (2.04-10.64)
Epilepsy	28	2.54 (1.69-3.81)	3.64 (1.7-7.81)	2.81 (0.95-8.33)	2 (1.14-3.51)
Learning disability - excluding Down's syndrome	58	1.08 (0.82-1.42)	1.87 (1.1-3.17)	0.8 (0.34-1.88)	0.92 (0.64-1.32)
Learning disability - Down's syndrome	5	2.45 (0.96-6.25)	2.69 (0.34-21.33)	NA	3.26 (1.13-9.4)
Fracture	39	1.41 (1.02-1.95)	1.51 (0.7-3.27)	1.37 (0.55-3.39)	1.41 (0.95-2.08)
Rare pulmonary diseases	9	5.04 (2.58-9.86)	2.77 (0.38-20.17)	10.34 (2.49-43.04)	4.91 (2.16-11.17)
Severe mental illness	6	1.43 (0.63-3.24)	3.94 (1.21-12.82)	1.88 (0.25-14.19)	0.69 (0.17-2.83)
Sickle cell disease	15	14.35 (8.48-24.28)	20.06 (7.15-56.27)	NA	16.41 (8.88-30.35)

Reference group is children without the condition. COVID-19 hospitalization is within 28 days of a positive test. HR: Hazard Ratio. CI: Confidence Interval. Hazard ratios were derived using cox proportional hazard model adjusting for age, sex, socioeconomic status, other risk groups of interest, and prior hospitalization. Alpha VOC became dominant by January 5, 2021 and Delta VOC became dominant by May 17, 2021.

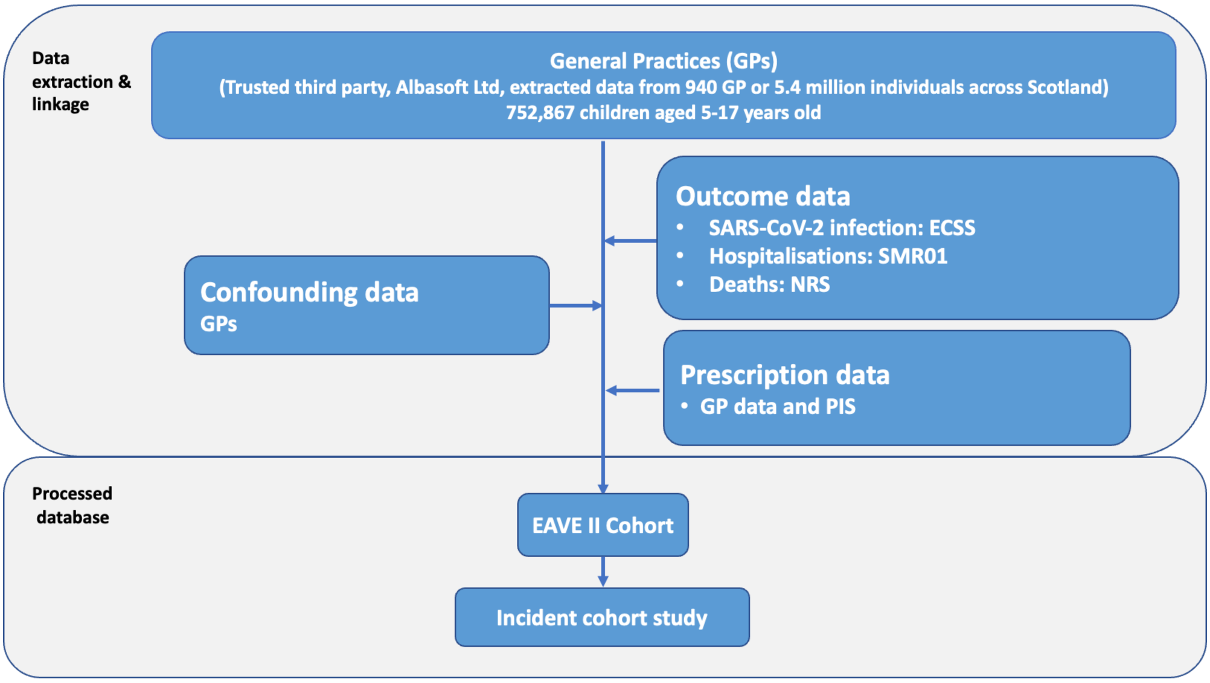

Community Health Index (CHI) numbers were used to link all datasets. ECSS: Electronic Communication of Surveillance in Scotland. SMR: Scottish Morbidity Record. NRS: National Records of Scotland. PIS: Prescribing Information System.

Figure S1: Data linkage diagram

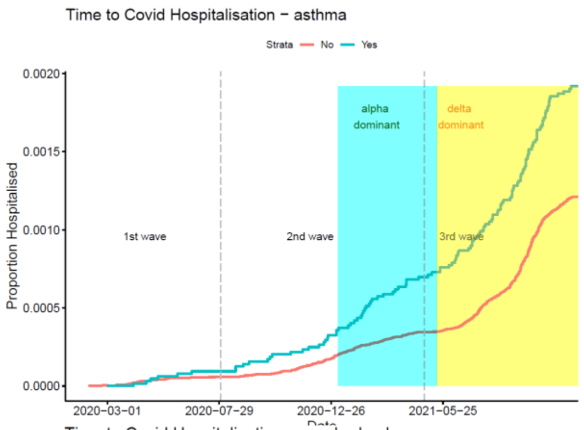
Time to Covid Hospitalisation − asthma
Strata No Yes
Proportion Hospitalised
0.0020
0.0015
0.0010
0.0005
0.0000
1st wave
2nd wave
3rd wave
alpha dominant
delta dominant
2020−03−01
2020−07−29
2020−12−26
2021−05−25

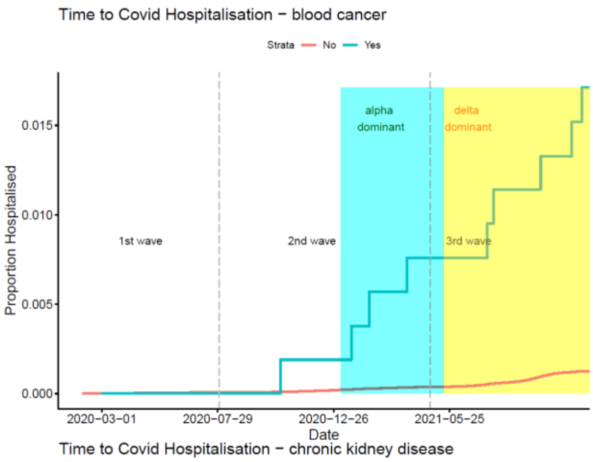
Time to Covid Hospitalisation − blood cancer
Strata No Yes
Proportion Hospitalised
0.015
0.010
0.005
0.000
1st wave
2nd wave
3rd wave
alpha dominant
delta dominant
2020−03−01
2020−07−29
2020−12−26
2021−05−25
Date

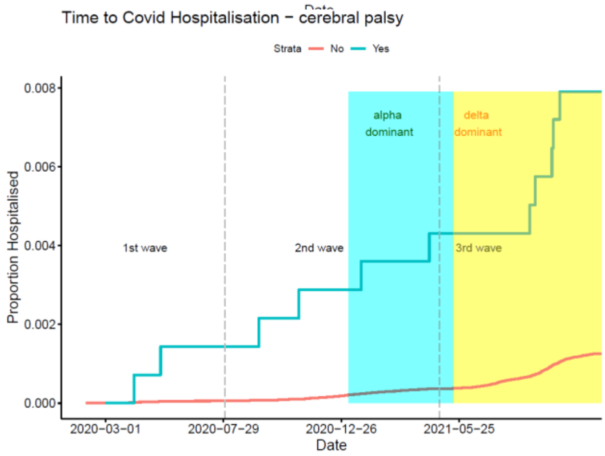
Time to Covid Hospitalisation − cerebral palsy
Strata No Yes
Proportion Hospitalised
0.008
0.006
0.004
0.002
0.000
1st wave
2nd wave
3rd wave
alpha dominant
delta dominant
2020−03−01
2020−07−29
2020−12−26
2021−05−25
Date

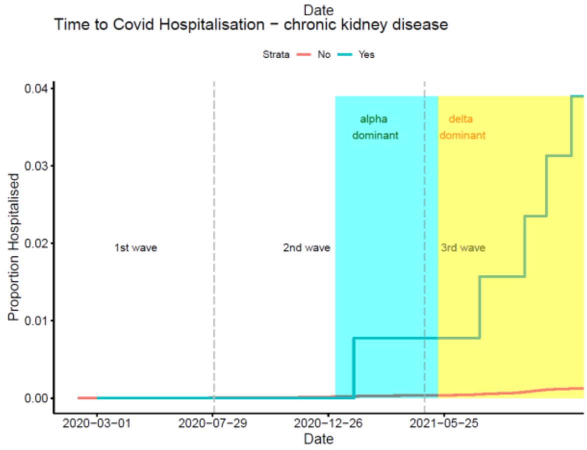
Time to Covid Hospitalisation − chronic kidney disease
Strata No Yes
Proportion Hospitalised
0.04
0.03
0.02
0.01
0.00
1st wave
2nd wave
3rd wave
alpha dominant
delta dominant
2020−03−01
2020−07−29
2020−12−26
2021−05−25
Date

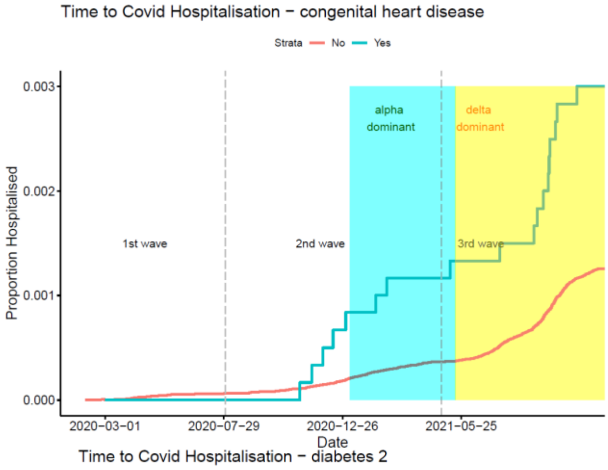
Time to Covid Hospitalisation − congenital heart disease
Strata No Yes
Proportion Hospitalised
0.003
0.002
0.001
0.000
1st wave
2nd wave
3rd wave
alpha dominant
delta dominant
2020−03−01
2020−07−29
2020−12−26
2021−05−25
Date

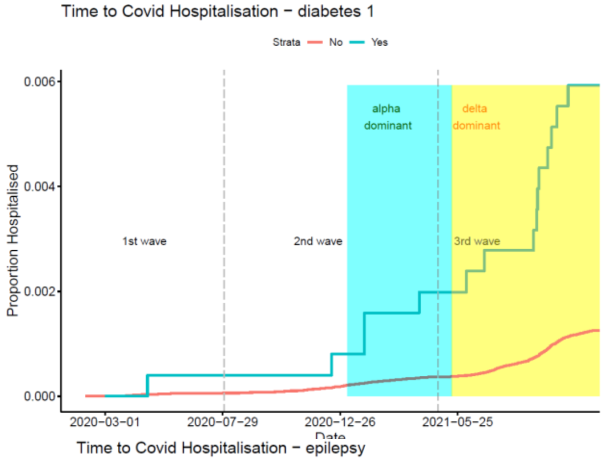
Time to Covid Hospitalisation − diabetes 1
Strata No Yes
Proportion Hospitalised
0.006
0.004
0.002
0.000
1st wave
2nd wave
3rd wave
alpha dominant
delta dominant
2020−03−01
2020−07−29
2020−12−26
2021−05−25

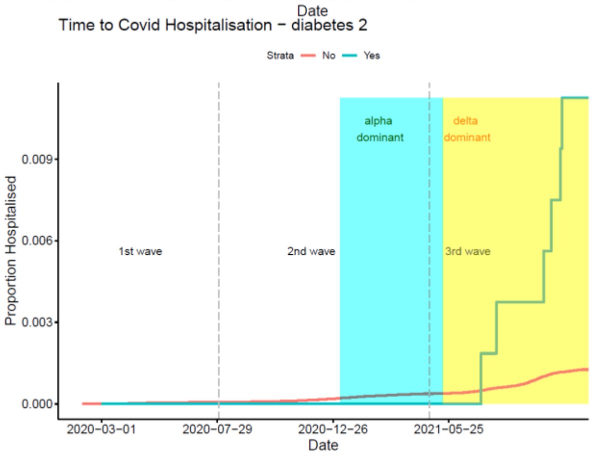
Time to Covid Hospitalisation − diabetes 2
Strata No Yes
Proportion Hospitalised
0.009
0.006
0.003
0.000
1st wave
2nd wave
3rd wave
alpha dominant
delta dominant
2020−03−01
2020−07−29
2020−12−26
2021−05−25
Date

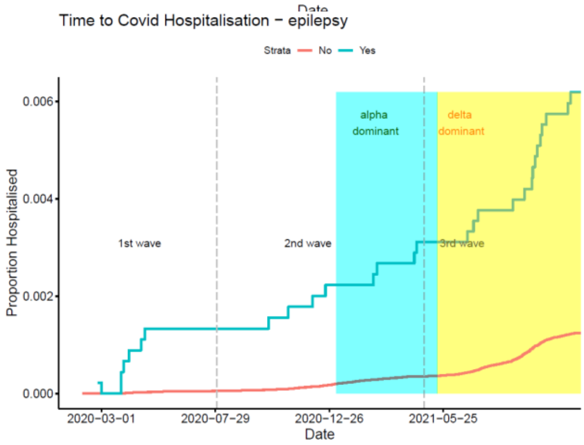
Time to Covid Hospitalisation − epilepsy
Strata No Yes
Proportion Hospitalised
0.006
0.004
0.002
0.000
1st wave
2nd wave
3rd wave
alpha dominant
delta dominant
2020−03−01
2020−07−29
2020−12−26
2021−05−25
Date

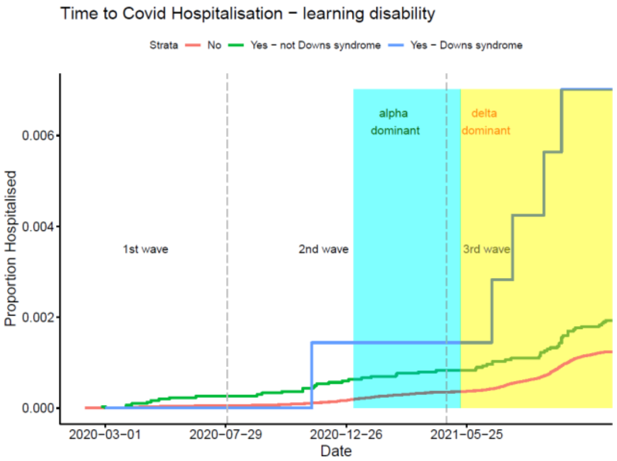
Time to Covid Hospitalisation − learning disability
Strata
No
Yes − not Downs syndrome
Yes − Downs syndrome
Proportion Hospitalised
0.000
0.002
0.004
0.006
1st wave
2nd wave
3rd wave
alpha dominant
delta dominant
2020−03−01
2020−07−29
2020−12−26
2021−05−25
Date

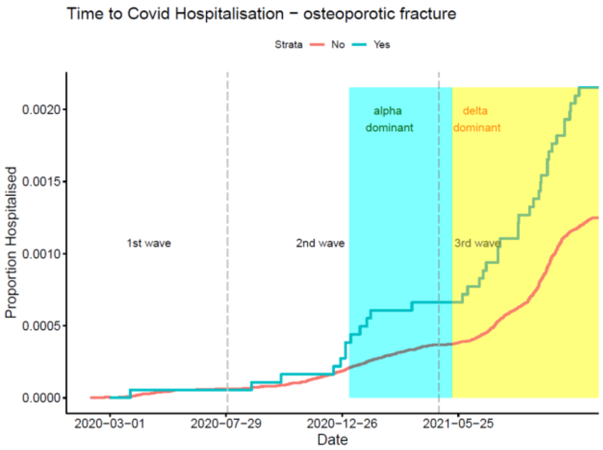
Time to Covid Hospitalisation − osteoporotic fracture
Strata
No
Yes
Proportion Hospitalised
0.0000
0.0005
0.0010
0.0015
0.0020
1st wave
2nd wave
3rd wave
alpha dominant
delta dominant
2020−03−01
2020−07−29
2020−12−26
2021−05−25
Date

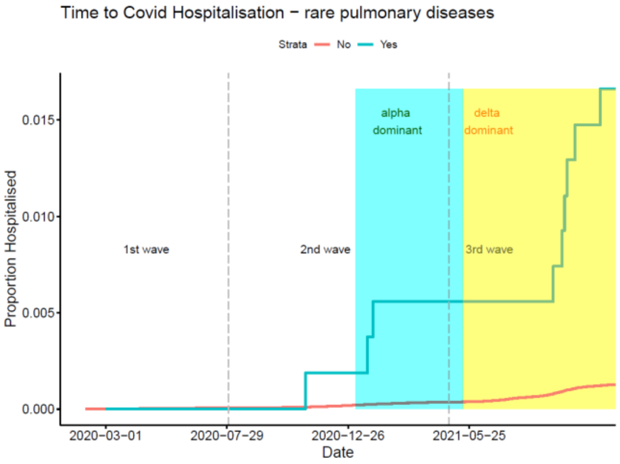
Time to Covid Hospitalisation − rare pulmonary diseases
Strata
No
Yes
Proportion Hospitalised
0.000
0.005
0.010
0.015
1st wave
2nd wave
3rd wave
alpha dominant
delta dominant
2020−03−01
2020−07−29
2020−12−26
2021−05−25
Date

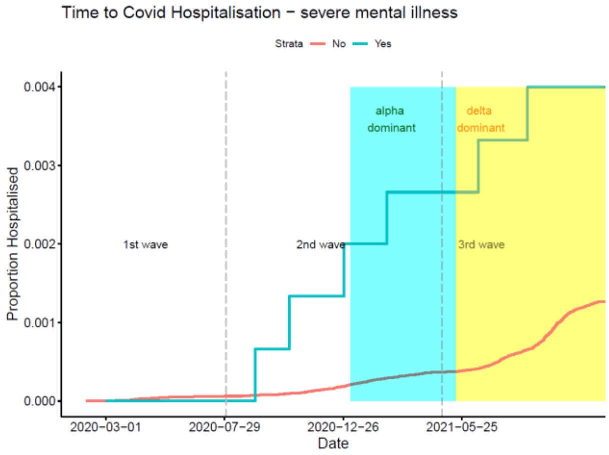
Time to Covid Hospitalisation − severe mental illness
Strata
No
Yes
Proportion Hospitalised
0.000
0.001
0.002
0.003
0.004
1st wave
2nd wave
3rd wave
alpha dominant
delta dominant
2020−03−01
2020−07−29
2020−12−26
2021−05−25
Date

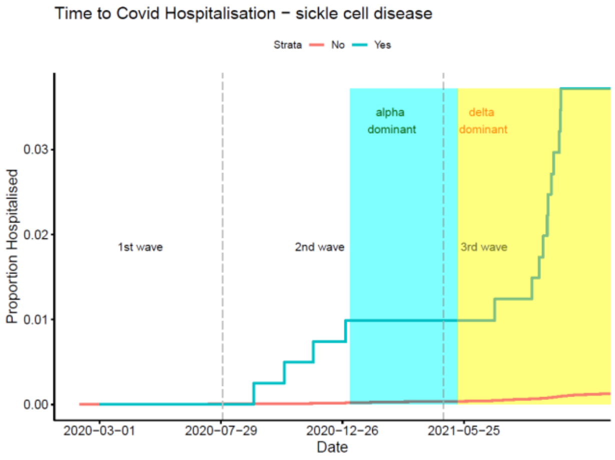
Time to Covid Hospitalisation − sickle cell disease
Strata
No
Yes
Proportion Hospitalised
0.00
0.01
0.02
0.03
1st wave
2nd wave
3rd wave
alpha dominant
delta dominant
2020−03−01
2020−07−29
2020−12−26
2021−05−25
Date

Figure S2: Time to COVID-19 hospitalization over time among children and young people aged 5 to 17 years old in each risk group of interest

The cumulative incidence of the outcome (COVID-19 hospitalization) was plotted against the time and stratified by the presence or absence of each risk condition. The cumulative incidence was estimated using the Kaplan-Meier method. The time period in the plots was marked by different waves (first wave from March to July 2020, second wave from August 2020 to April 2021 and third wave from May to July 2021) and also by different dominant variants. Alpha variant of concern (VOC) became dominant by January 4, 2021 and Delta VOC became dominant by May 17, 2021. These curves generally show an increasing difference between children in a risk group and those not in the risk group from the second wave onwards.

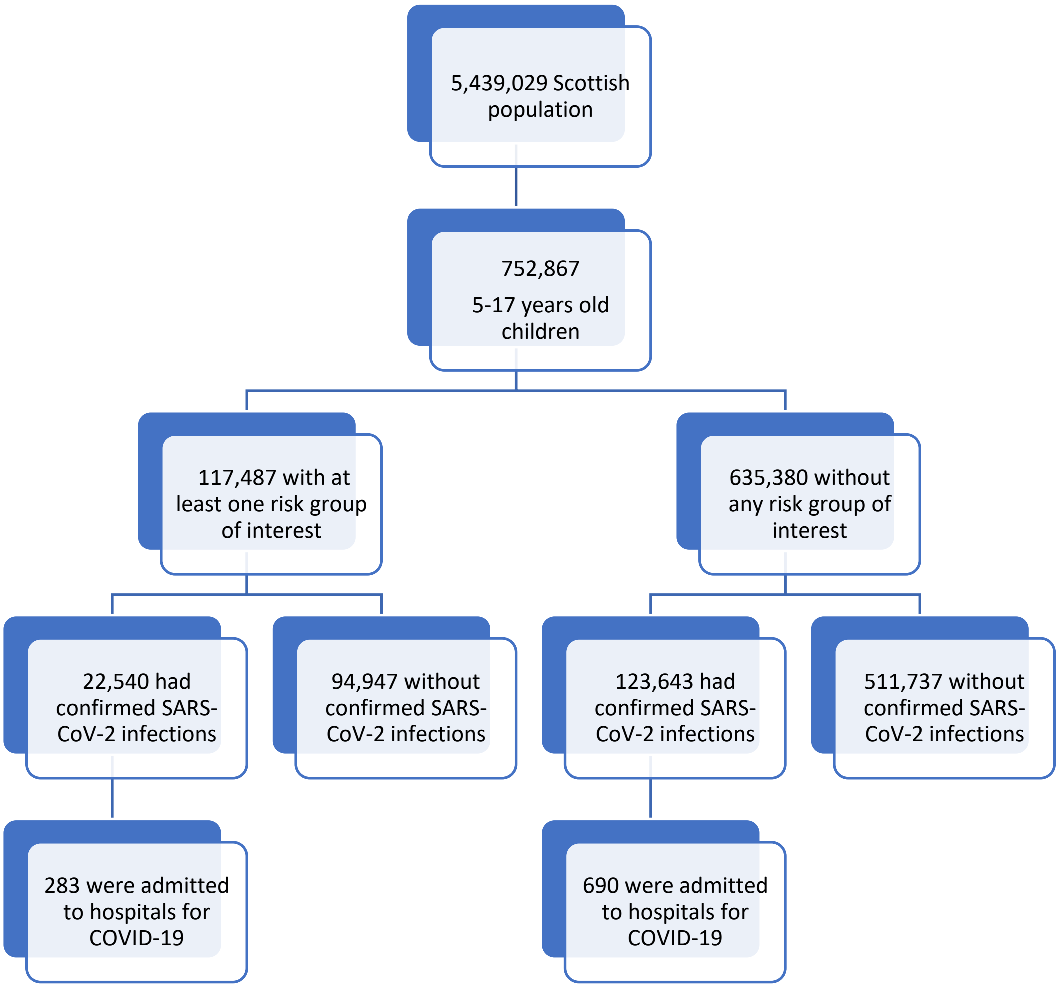

Figure S3: A flow chart of study population

157

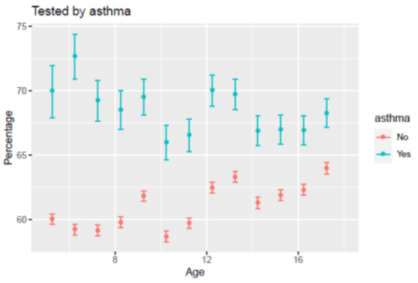
Tested by asthma
Percentage
Age
asthma
No
Yes

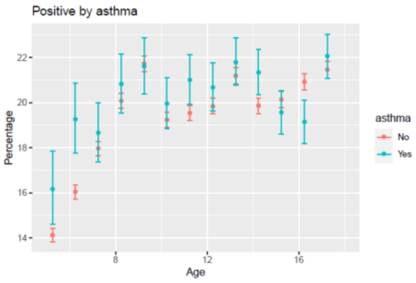
Positive by asthma
Percentage
Age
asthma
No
Yes

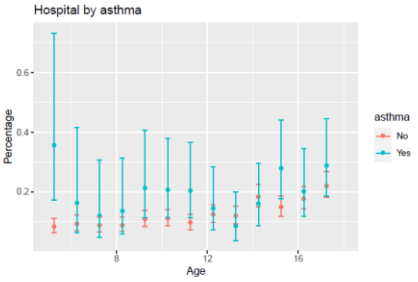
Hospital by asthma
Percentage
Age
asthma
No
Yes

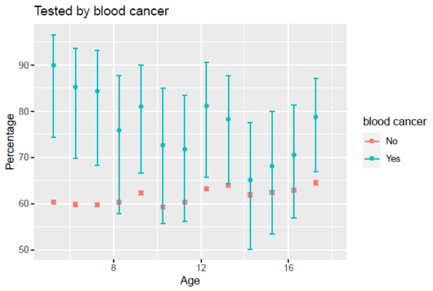
Tested by blood cancer
Percentage
Age
blood cancer
No
Yes

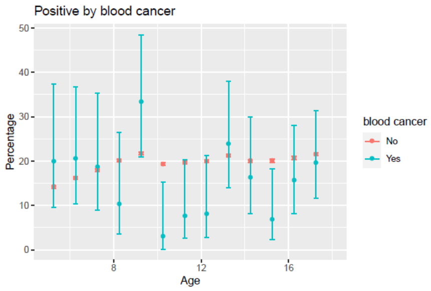
Positive by blood cancer
Percentage
Age
blood cancer
No
Yes

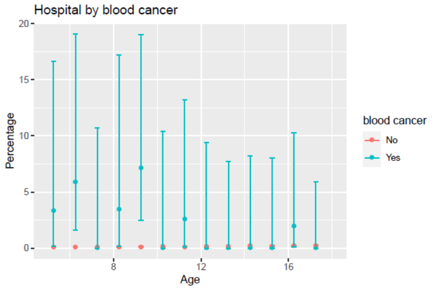
Hospital by blood cancer
Percentage
Age
blood cancer
No
Yes

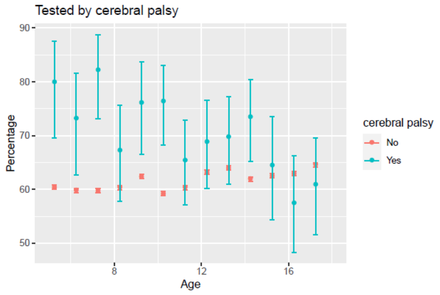
Tested by cerebral palsy
Percentage
Age
cerebral palsy
No
Yes

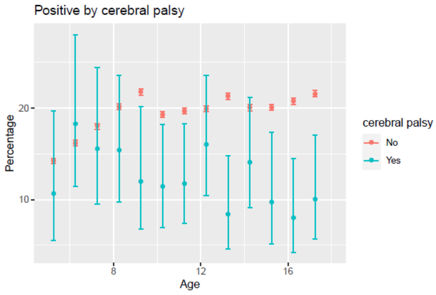
Positive by cerebral palsy
Percentage
Age
cerebral palsy
No
Yes

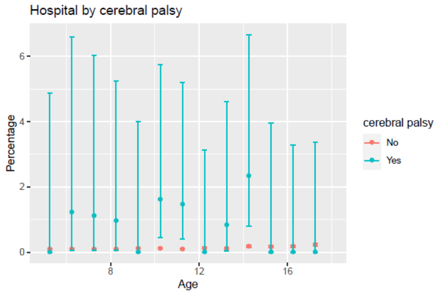
Hospital by cerebral palsy
Percentage
Age
cerebral palsy
No
Yes

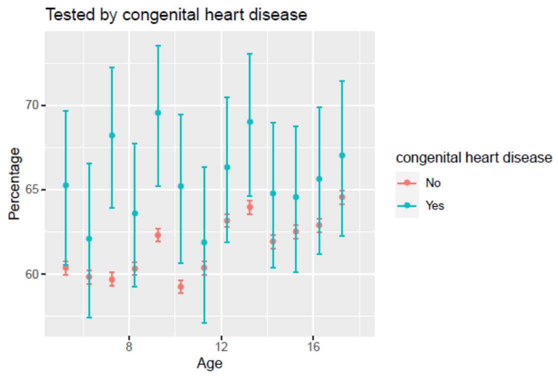
Tested by congenital heart disease
Percentage
Age
congenital heart disease
No
Yes

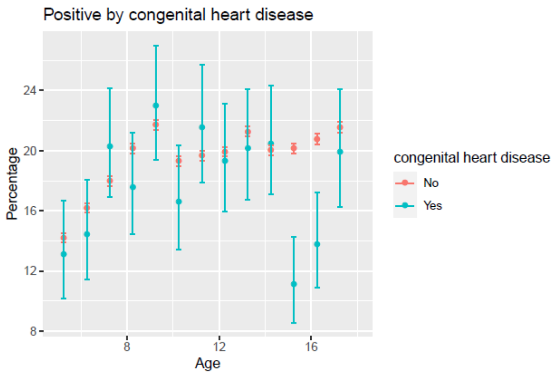
Positive by congenital heart disease
Percentage
Age
congenital heart disease
No
Yes

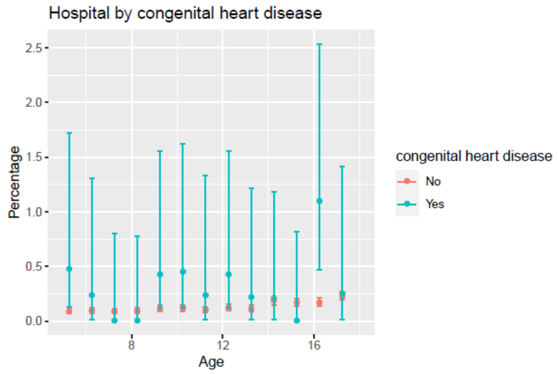
Hospital by congenital heart disease
Percentage
Age
congenital heart disease
No
Yes

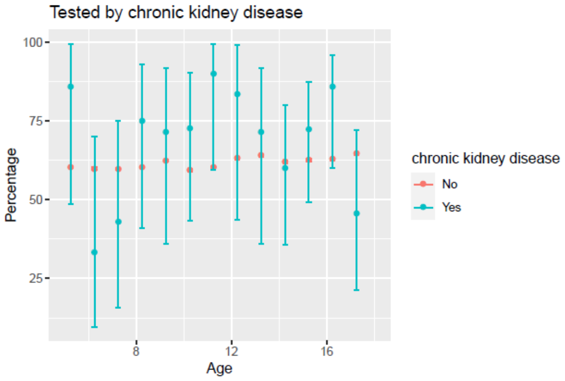
Tested by chronic kidney disease
Percentage
Age
chronic kidney disease
No
Yes

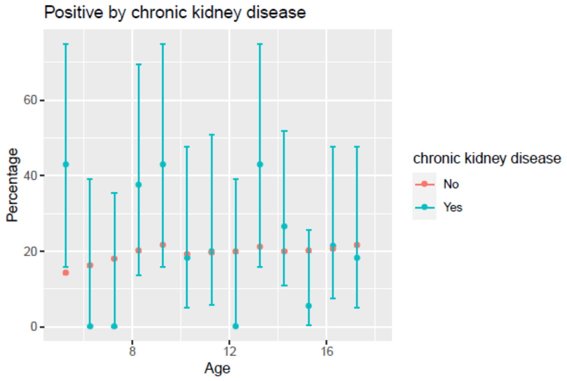
Positive by chronic kidney disease
Percentage
Age
chronic kidney disease
No
Yes

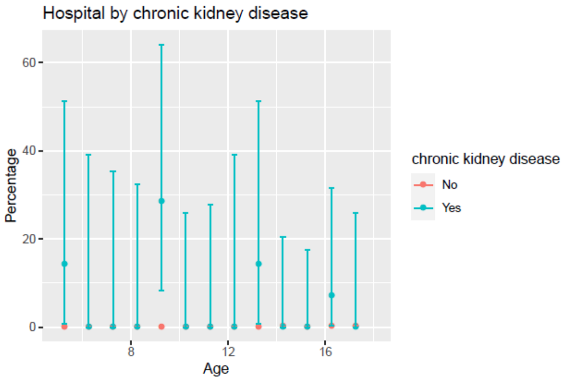
Hospital by chronic kidney disease
Percentage
Age
chronic kidney disease
No
Yes

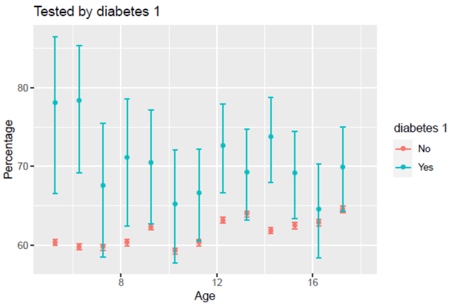
Tested by diabetes 1
80
70
60
Percentage
8
12
16
Age
diabetes 1
No
Yes

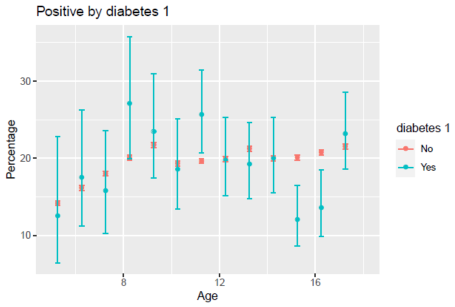
Positive by diabetes 1
30
20
10
Percentage
8
12
16
Age
diabetes 1
No
Yes

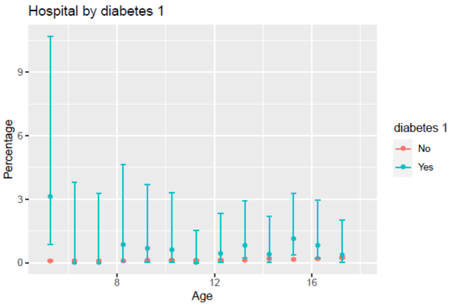
Hospital by diabetes 1
9
6
3
0
Percentage
8
12
16
Age
diabetes 1
No
Yes

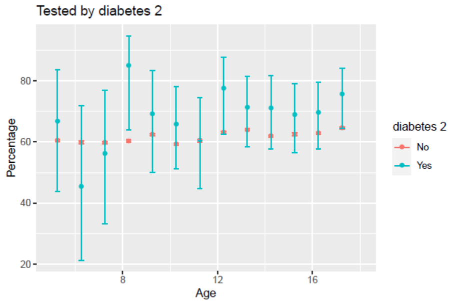
Tested by diabetes 2
80
60
40
20
Percentage
8
12
16
Age
diabetes 2
No
Yes

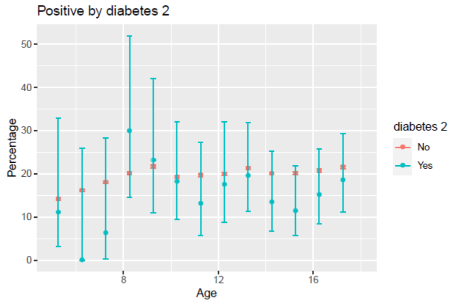
Positive by diabetes 2
50
40
30
20
10
0
Percentage
8
12
16
Age
diabetes 2
No
Yes

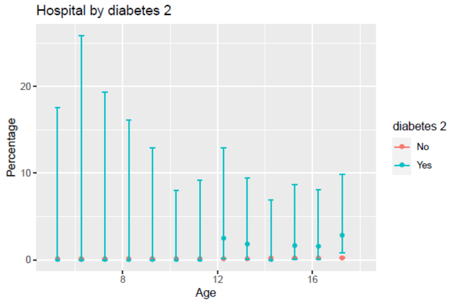
Hospital by diabetes 2
20
10
0
Percentage
8
12
16
Age
diabetes 2
No
Yes

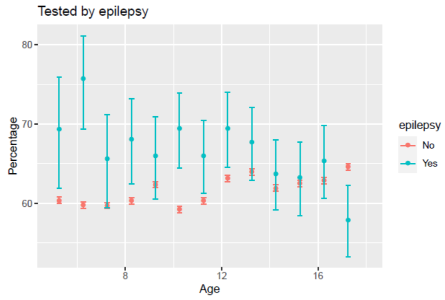
Tested by epilepsy
80
70
60
Percentage
8
12
16
Age
epilepsy
No
Yes

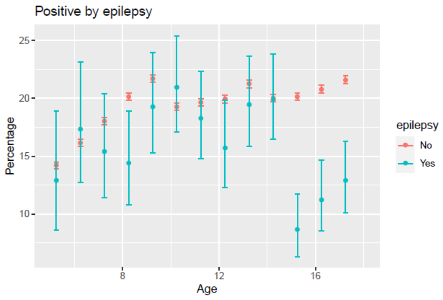
Positive by epilepsy
25
20
15
10
Percentage
8
12
16
Age
epilepsy
No
Yes

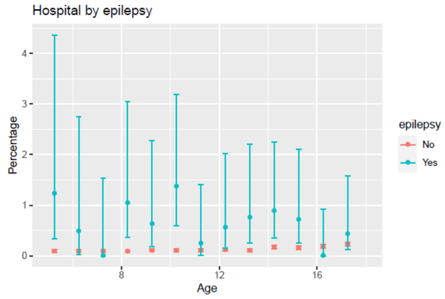
Hospital by epilepsy
4
3
2
1
0
Percentage
8
12
16
Age
epilepsy
No
Yes

160

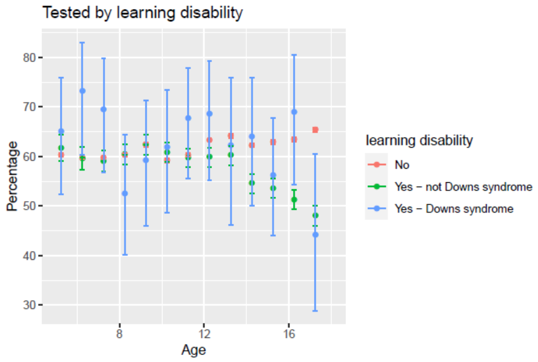
Tested by learning disability
Percentage
80
70
60
50
40
30
8
12
16
Age
learning disability
No
Yes – not Downs syndrome
Yes – Downs syndrome

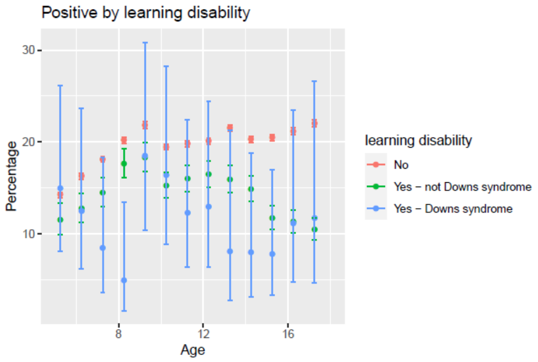
Positive by learning disability
Percentage
30
20
10
8
12
16
Age
learning disability
No
Yes – not Downs syndrome
Yes – Downs syndrome

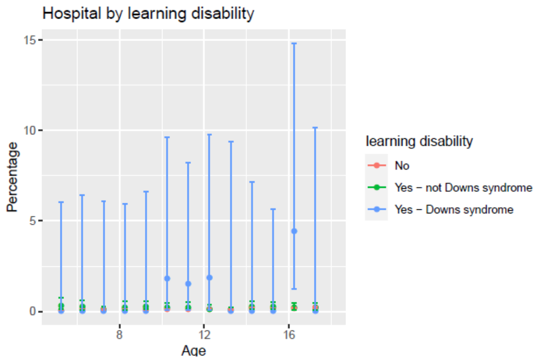
Hospital by learning disability
Percentage
15
10
5
0
8
12
16
Age
learning disability
No
Yes – not Downs syndrome
Yes – Downs syndrome

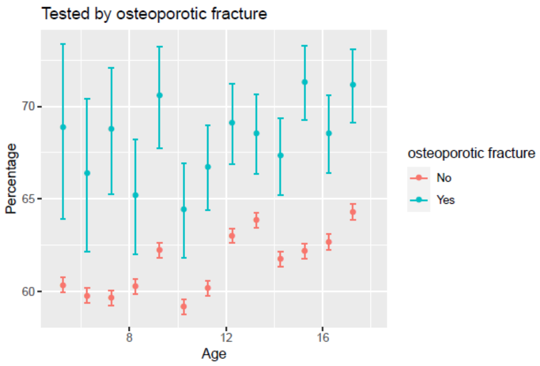
Tested by osteoporotic fracture
Percentage
70
65
60
8
12
16
Age
osteoporotic fracture
No
Yes

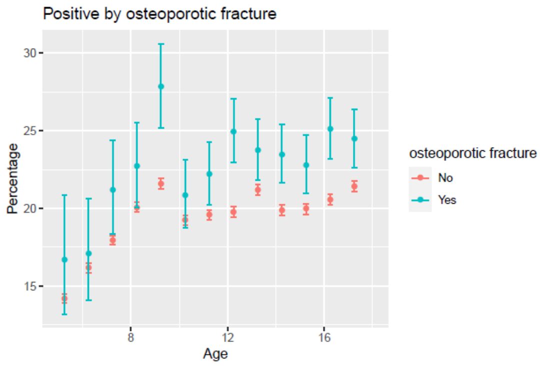
Positive by osteoporotic fracture
Percentage
30
25
20
15
8
12
16
Age
osteoporotic fracture
No
Yes

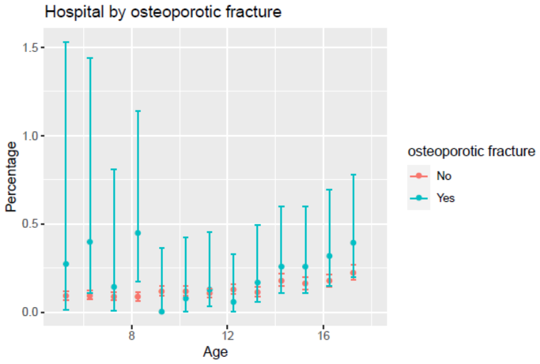
Hospital by osteoporotic fracture
Percentage
1.5
1.0
0.5
0.0
8
12
16
Age
osteoporotic fracture
No
Yes

Tested by rare pulmonary diseases

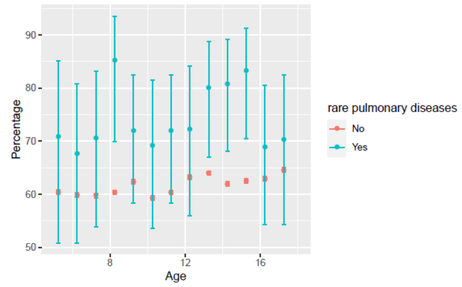

Positive by rare pulmonary diseases

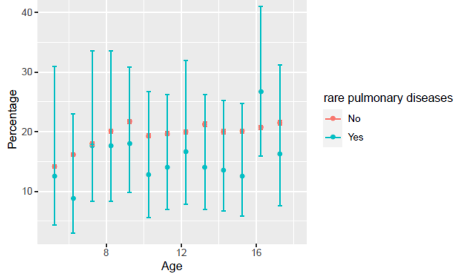

Hospital by rare pulmonary diseases

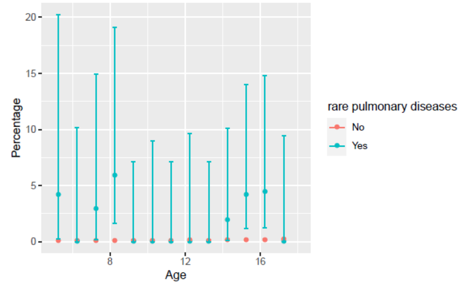

Tested by severe mental illness

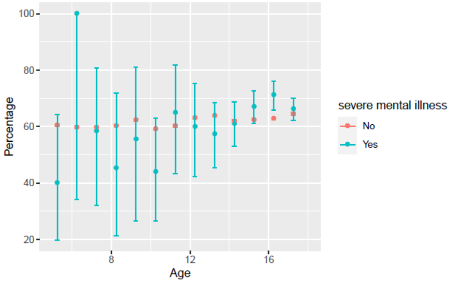

Positive by severe mental illness

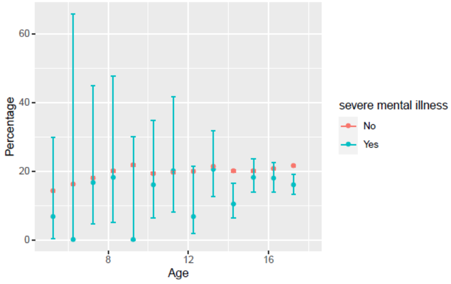

Hospital by severe mental illness

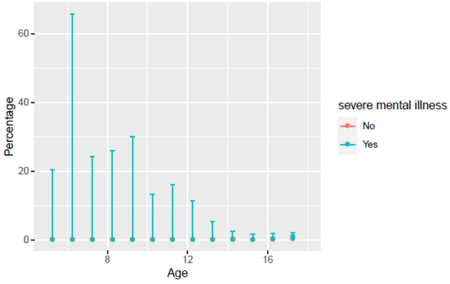

Tested by sickle cell disease

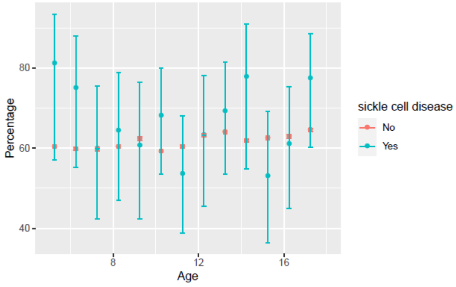

Positive by sickle cell disease

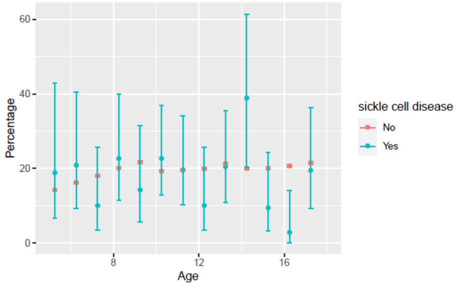

Hospital by sickle cell disease

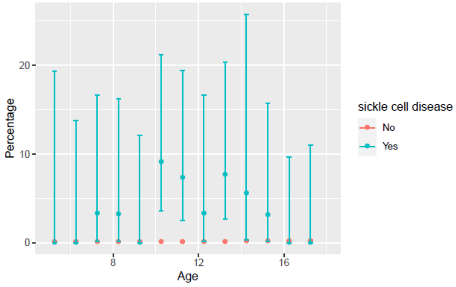

Figure S4: Age pattern for 5-17 years old with each risk condition of interest and percentage of being tested, test positive and COVID-19 hospitalization over age in each risk group of interest

These plots show the percentage of children in each age group (at March 2020) who were tested, tested positive and admitted to hospital with COVID-19 separately for those with the specified condition and those without. The confidence intervals are calculated using Wilson's method. The number hospitalized are small and the confidence intervals wide. There is more precision in the proportions tested and testing positive. Generally, these charts show that children in the risk group are more likely to be tested, have similar levels of testing positive but are more likely to be admitted to hospital.

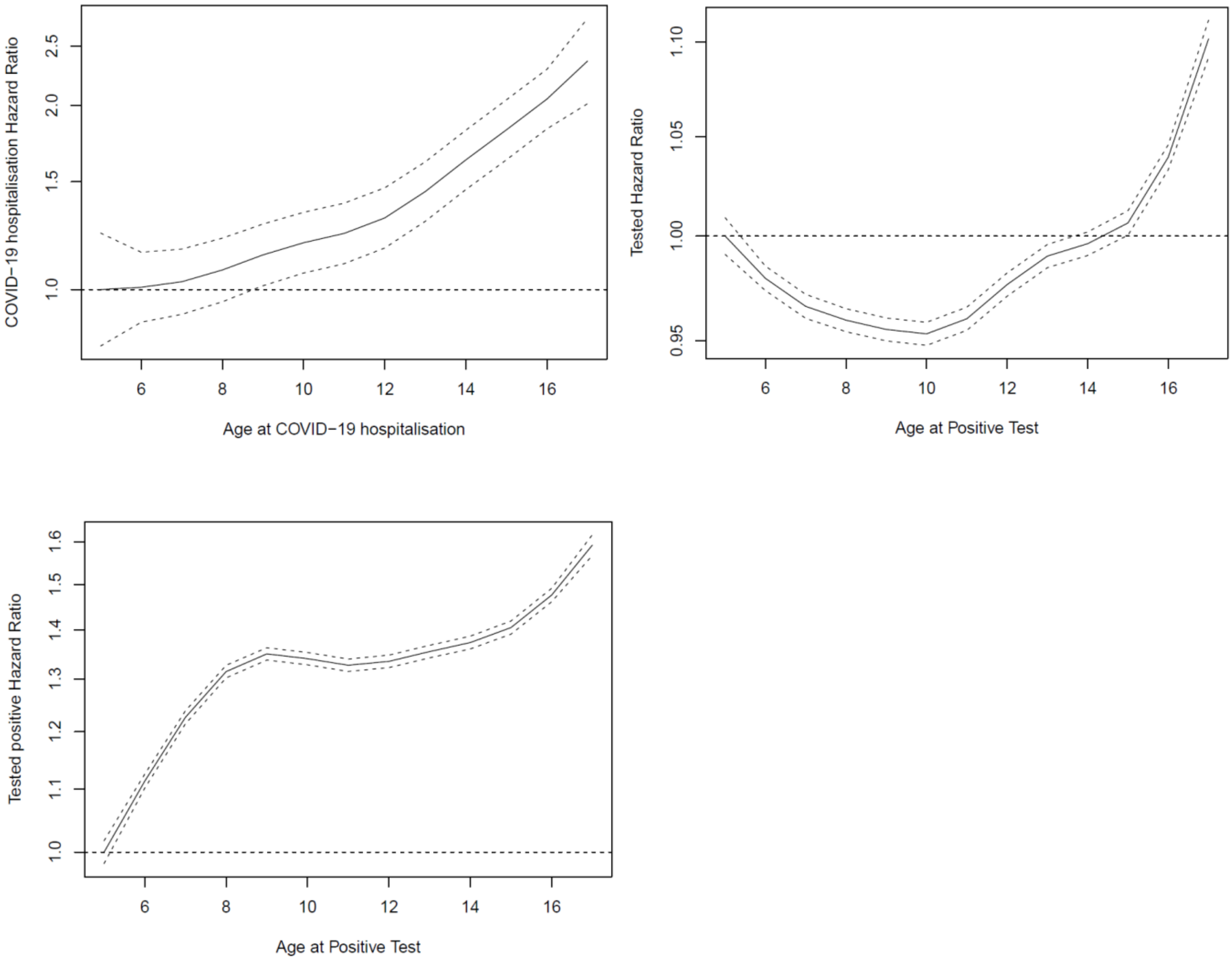

Figure S5: Unadjusted non-linear age effect on COVID-19 hospitalization (penalised splines were used in the statistical modelling)
